# Japanese urban household carbon footprints during early-stage COVID-19 pandemic were consistent with those over the past decade

**DOI:** 10.1038/s42949-023-00095-z

**Published:** 2023-03-29

**Authors:** Yin Long, Yoshikuni Yoshida, Yida Jiang, Liqiao Huang, Wentao Wang, Zhifu Mi, Yosuke Shigetomi, Keiichiro Kanemoto

**Affiliations:** 1 Graduate School of Engineering, University of Tokyo, 7-3-1 Hongo, Bunkyo-ku, Tokyo, 113-8654 Japan; 2 Graduate Program in Sustainability Science - Global Leadership Initiative, The University of Tokyo, 5-1-5 Kashiwanoha, Kashiwa, Chiba, 277-8563 Japan; 3 The Administrative Center for China’s Agenda 21, No. 8 Yuyuan Nan Road, Haidian District, Beijing, China; 4 The Bartlett School of Sustainable Construction, University College London, London, WC1E 7HB UK; 5 Faculty of Environmental Science, Nagasaki University, 1-14 Bunkyo-machi, Nagasaki, 852-8521 Japan; 6 Research Institute for Humanity and Nature, 457-4 Motoyama, Kamigamo, Kita-ku, Kyoto, 603-8047 Japan; 7 Graduate School of Environmental Studies, Tohoku University, Aoba, 468-1, Aramaki, Aoba-ku, 980-8572 Sendai, Japan

**Keywords:** Environmental impact, Climate-change mitigation, Climate-change policy

## Abstract

As urbanization accelerates worldwide, substantial energy and services are required to meet the demand from cities, making cities major contributors to adverse environmental consequences. To bridge the knowledge gap in the absence of fine-grained city-level climate protection measures due to data availability and accuracy, this study provides a detailed carbon emission inventory for analyzing the monthly fluctuations based on citizens’ daily consumption behaviors. Here, carbon emissions embodied in approximately 500 household consumption items were calculated in 47 prefectural-level cities in Japan from 2011 to June 2021. We analyzed the results considering the regional, seasonal, demand, and emission way-specific aspects, and compared the emission before and during the COVID-19 pandemic. Notably, the carbon footprints during the pandemic were consistent with the previous level despite downtrends in specific categories. This study provides an example of utilizing city-level emission data to improve household green consumption behavior as references for enriching city-level decarbonization paths.

## Introduction

Urban areas, including cities and metropolitan areas, are the drivers of economic growth, contributing nearly 80% of the global GDP and consuming more than 60% of the resources^1^ as the majority of the world population congregates in urban areas^2^. At the same time, world-scale urbanization has been proven to be one of the main reasons behind the global greenhouse gas (GHG) emission increase^3–5^. In fact, cities are demonstrated to have contributed approximately 70% of the world’s total carbon emissions^1,6,7^. Therefore, cities play an important role in the implementation of world-scale decarbonization measures. As a response to alleviate the climatic issues raised by anthropogenic activities, the United Nations released the ambitious 2030 Sustainable Development Agenda, setting out 17 sustainable development goals (SDGs) to guide future global development^8^. Cities are involved in multiple SDGs (e.g., Goal 11: Sustainable cities and communities, Goal 12: Responsible Consumption and Production), emphasizing the need to build inclusive, disaster-resilient, and sustainable cities. Furthermore, increasing focus has also been drawn to city-level adaptation plans or customizing city-specific emission reduction actions, which is the focal point in the New Urban Agenda^9^ and the C40 Cities Climate Leadership Group^10^.

Mindful of both its pivotal economical role and the huge emission reduction potential, cities are requested to act as decarbonization pioneers to lead the world in terms of climate change adaptation, governing measures innovation, and long-term sustainable paths evolution^11,12^. However, before adopting measures to realize the listed goals, it is essential to quantify the emissions accurately, so as to discern emission-intensive activities, enact effective emission reduction plans and avoid unclear and inefficient efforts^13,14^. Among the different sectors of national accounts, the largest consumption terminal is found to be the household sector^15,16^, particularly in urban areas^17–19^. Previous studies indicate that the household sector accounts for 70% of the total GHG emissions embodied in final consumption from the results of consumption-based carbon accounting^20–22^. In terms of sources of household emissions, the demand for other goods (e.g., food and clothing) and services (e.g., medical care, education, recreation) generates emissions indirectly in residential life^18^. In fact, indirect ways of household consumption (carbon emission embodied in daily goods and service consumption) are more emission-intensive than direct energy consumption (e.g., transportation, cooking, heating appliances, etc.). Therefore, to promote efficient emission reduction and achieve carbon neutrality goals, analyzing household consumption behaviors and the associated carbon emission in the entire supply chain (i.e., carbon footprint) is of great importance.

Currently, how to capture or decrease urban household carbon emission is still under discussion, with particular focus on the definition of geographic and system boundaries, and developing corresponding countermeasures^23,24^. For example, the environmental impact embodied in economic interactions has been widely discussed at the national level, such as in Japan^25,26^, China^27,28^, and the US^29,30^. On top of that, the recent discussion also focuses on the subnational level such as prefectures and cities, such as Beijing (China)^31–34^, the Tokyo metropolitan area^35^ and other prefectural cities in Japan^36,37^, and several Australian cities^38,39^. The listed research provides essential examples of the applicability of carrying out urban-scale household carbon accounting. Nevertheless, the research period of existing studies on city-level household carbon accounting is often limited to a fixed time point, and there is a lack of sufficient time-series data to reveal the changes in the household carbon footprint over time^18,40–42^. It is therefore extremely difficult to analyze the spatial-temporal changes in carbon footprints in response to unexpected socio-economic events such as COVID-19.

In the context of this research gap, the present study generates city-level carbon footprint data from 2011 to 2021 to quantify both the direct and indirect city-level carbon footprints and takes 47 Japanese cities as an example. The cities selected in this study are the capital cities of 43 prefectures, two urban prefectures (Osaka and Kyoto), one territory (Hokkaidō), and the metropolis Tokyo, the population of which accounts for more than 50% of Japan’s population^43^. Approximately 500 household consumption items (by month/city) were captured with their embodied carbon footprint, covering citizens’ major living demands (e.g., food, home energy, accommodation, and transportation). To note, the monthly carbon footprint is calculated based on the annual input–output table and monthly household consumption data. In addition, the emission variations from January 2020 were extracted, with a special focus on revealing how the consumption behaviors of city residents were impacted in response to COVID-19. The findings provide important insights for evaluating the environmental consequences of citizen behaviors under the abrupt impact of unexpected social events.

## Results

### City-level household carbon footprint variance from 2011 to 2021

**Fig. 1:**
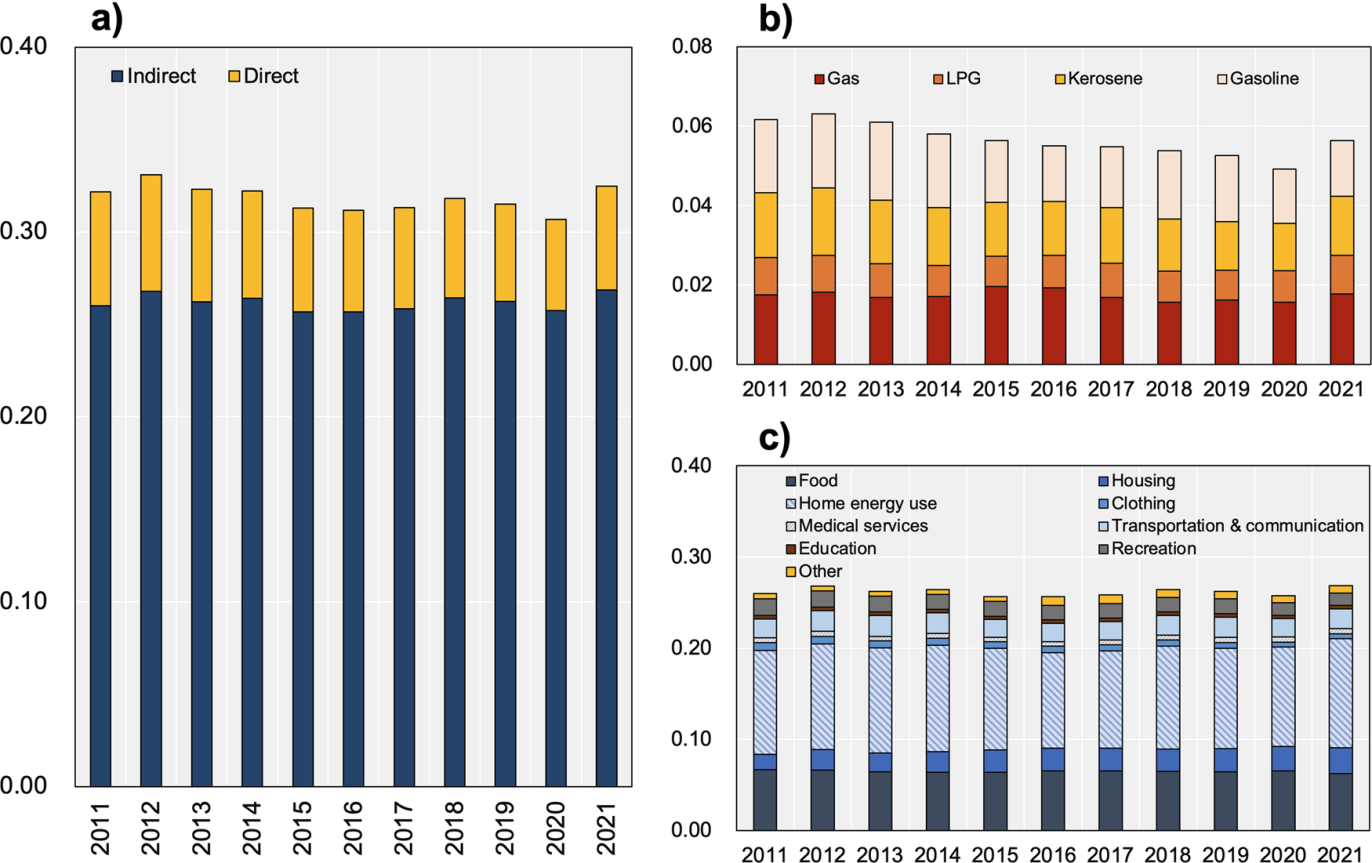
The results of monthly household carbon footprints (in tCO_2_eq/cap/month). **a** Monthly total, **b** Direct, and **c** Indirect carbon footprint.

In Fig. 1, the results of the monthly average carbon emissions in 47 Japanese cities since 2011 are presented, and details on (a) the total amount of direct and indirect emissions, (b) the content of direct emissions by fuel type, and (c) indirect emissions by household expenditure type are separately provided in the subgraphs. By the time of writing, the data for 2021 were not available after July, and the monthly average emissions from January to June are adopted to represent the annual value of 2021. Over the 11 years, the trend of total carbon emissions in Japan fluctuated, with indirect emissions accounting for more than 80% and direct emissions constituting only a small fraction.

The results of direct emissions of Japanese cities indicate that gas and gasoline consumption are the primary sources of direct carbon emissions among the four types of fuels, followed by kerosene and LPG. For indirect emissions, home energy use and food are responsible for more than half of the two main categories, and transportation and communication, housing, and recreation are the categories with relatively high emissions. In contrast, emissions induced by clothing, medical services, education, and the ‘other’ categories account for only a small portion of indirect emissions.

It should be stressed that the emission levels for 2021 were generally higher, which can be explained by the higher energy consumption in the first quarter of the year (refer to Supplementary Fig. 1). In terms of total emissions, the monthly average emissions of prefectural-level cities in Japan declined from the peak value of 0.331 tCO_2_eq/cap in 2012, reaching a minimum value of 0.312 tCO_2_eq/cap in 2016. Subsequently, the total emissions rebounded, reaching a value of 0.318 tCO_2_eq/cap in 2018, and then declined in the following years. Due to COVID-19, the total emissions fell to the lowest level in the past decade in 2020, reaching a minimum value of 0.307 tCO_2_eq/cap. Direct emissions showed a discernible downward trend, notwithstanding the limited change in the energy consumption structure.

By exploring the contribution ratio of emissions from different fuel types to direct emissions, a slight increase in the share of gas and gasoline and a decrease in the share of LPG and kerosene were observed. According to our results, indirect emissions fluctuated from year to year, which approximately matched the trend of total emissions since indirect emissions determined the overall trend. Therefore, changes in emissions due to different household expenditure types of help explain variations in total emissions. In addition to the annual variations, Supplementary Fig. 1 provides the direct and indirect emission results for each month, highlighting the seasonal trends. Notably, a strong seasonal trend was observed in direct emissions, which peaked during the winter and bottomed in the summer. Additional findings can be found in the Supplementary Information.

### Spatial distribution of emission by types

**Fig. 2:**
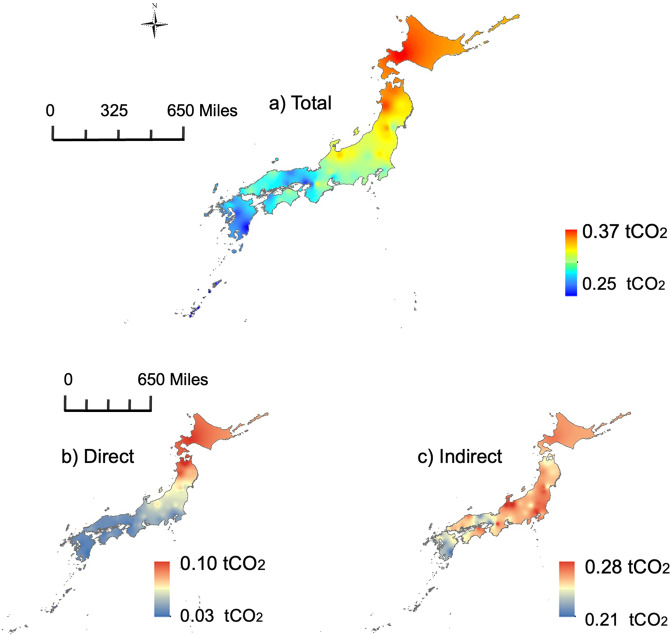
Interpolation results of the distribution of household carbon footprint in Japanese cities. **a** Total, **b** Direct, and **c** Indirect carbon footprints (in tCO_2_eq/cap/month).

The distribution of total, direct, and indirect carbon emissions in 47 major cities in Japan is shown in Fig. 2. The spatial distribution map of total emissions in all major cities in Japan demonstrates that per capita emissions are higher in the northeast regions and lower in the southwest regions, relating to the climate and heating needs due to the latitudes of cities, and the distribution map of direct emissions shows a similar pattern. However, the intensity of indirect emissions did not exhibit a gradual decline from north to south spatially. Although indirect emissions in the north are still higher than those in the south, cities with the highest emission levels appear in metropolitan areas, including Tokyo and the surrounding prefectures (Saitama, Chiba, and Kanagawa), Nagoya, Osaka, Kyoto, and cities in the northern part of Honshu Island. In contrast, the southeastern coastal areas had the lowest emissions. The average monthly carbon footprints each year from 2011 to 2020 are demonstrated in the form of boxplots in Supplementary Fig. 2, with cities arranged from north to south. In addition, Supplementary Fig. 3 and Supplementary Fig. 4 show the constituents of the direct and indirect emissions in all 47 cities. Significant differences exist in the major sources of emissions across the cities for direct emissions. In general, cities with higher kerosene and LPG consumption had higher direct emissions. For instance, northeastern cities (e.g., Sapporo, Aomori, Morioka, and Akita) usually rely on kerosene as the dominant energy source, contributing to more than half of the emissions. In contrast, gas and gasoline occupy dominant positions in the energy consumption structure of the southern cities (e.g., Naha, Miyazaki, Kumamoto, and Matsuyama), which emit relatively fewer GHGs. To visualize the impact of the energy consumption structure, Supplementary Fig. 5 in the appendix presents the proportion of direct emissions by fuel type, and the cities are arranged according to kerosene consumption.

To clarify the categories of consumption that induce carbon emissions and show their geographical differences, we decomposed the carbon footprints according to the final demand. Here, we classify the final demands into nine major categories; the main content of most categories can be explained by their names, while some cannot be discerned by their names alone. For instance, the housing category contains emissions due to housing, household furniture, and equipment, whereas the home energy use sector covers emissions produced by household energy demand, such as electricity and gas. In addition, the transportation and communication category includes all types of travel, whether public or private transport. The ‘other’ category includes emissions due to tobacco, religious tributes, weddings and funerals, and non-savings insurance.

**Fig. 3:**
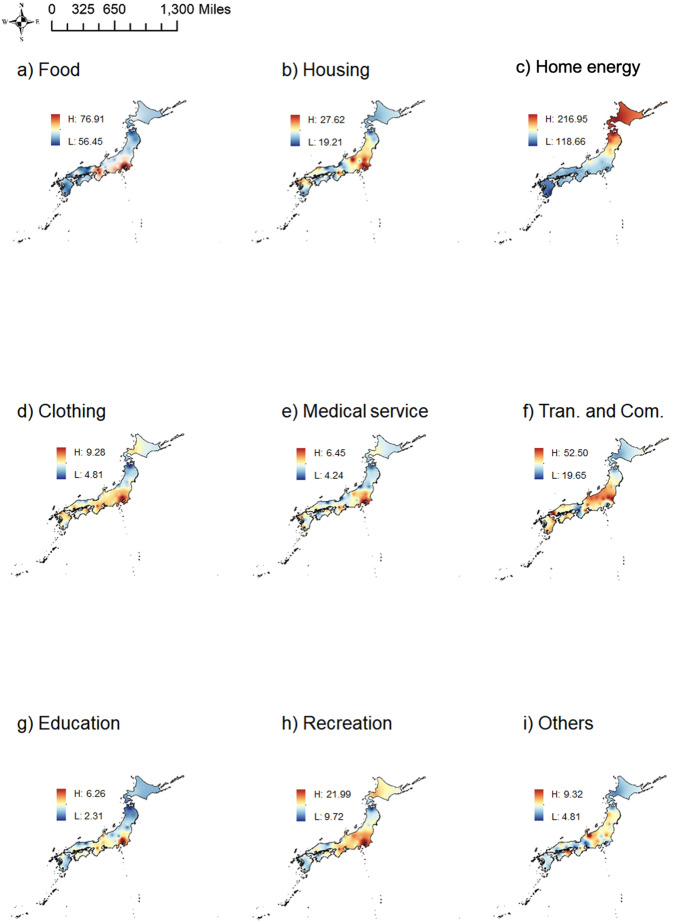
Distribution of city-level carbon footprints by demand (in kgCO_2_eq/cap/month). The categories of household consumption demand include: **a** Food, **b** Housing, **c** Home energy, **d** Clothing, **e** Medical service, **f** Transportation and Communication, **g** Education, **h** Recreation, and **i** Others.

The distributions of household carbon footprints in the nine categories across the 47 cities of Japan were generated by Inverse Distance Weighted (IDW) interpolation, as shown in Fig. 3, which is the monthly average results from 2011 to 2021. First, for carbon footprints driven by the demand for food, cities with higher emissions are concentrated in metropolitan areas (Tokyo, Chiba, Yokohama, and Saitama) and other large cities (Osaka, Kyoto, and Nagoya) due to frequent eating out and the relatively large share of meat and beverages, such as alcohol, in people’s diets. The emissions from home energy use demand show strong geographical characteristics, with the northeast region, which has higher heating needs, emitting more GHGs, and the southern region presenting lower per capita emissions. In terms of housing, cities with high emissions are more dispersed; the city with the highest emissions is Nara, followed by Nagano, and cities in the capital region. For clothing, medical care, education, and recreation, the same characteristics of high emissions in large cities (metropolitan area, Osaka) are present, while the Tohoku region shows low emissions. Although large cities are commonly perceived to have high demands for transportation and communication, the study results show that large cities, such as Tokyo and Osaka, possess the lowest emission results in the country. By analyzing the detailed data, we explored the reasons behind this and found that more convenient public transportation is available and parking fees are high in large cities, thereby reducing the use of private vehicles and fuel consumption. Hokkaido and Tohoku cities also produced fewer emissions in this demand category. For the ‘other’ category, the needs in different cities vary and need to be discussed separately. More details regarding the carbon footprints of the 47 cities by demand are provided in Supplementary Fig. 6. This figure indicates which specific cities generate larger emissions in different demand categories and can be regarded as a supplement to Supplementary Fig. 3.

### Response to COVID-19 at the city level (household)

**Fig. 4:**
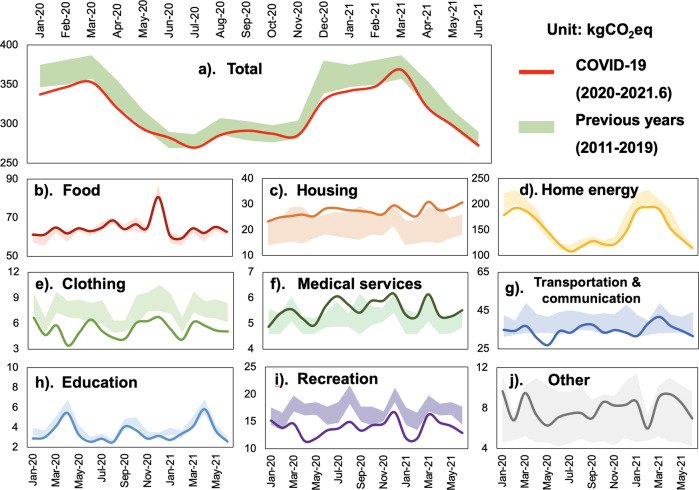
City-level carbon footprints based on household demand (in kgCO_2_eq/cap/month) during the COVID-19 pandemic. The household carbon footprints in the following demand categories: **a** Total, **b** Food, **c** Housing, **d** Home energy, **e** Clothing, **f** Medical service, **g** Transportation and communication, **h** Education, **i** Recreation, and **j** Others, respectively.

To explore the changes in household carbon footprints during the pandemic, we compared the monthly carbon footprints since 2020 with the corresponding monthly footprints in the past nine years, as Japan’s city-level household carbon footprints possesses seasonal features. In Fig. 4, the average carbon footprints of prefectural-level cities in Japan from January 2020 to June 2021 are provided, including the results of total emissions and decomposed demand-driven emissions. The carbon footprints during the pandemic are expressed by lines, and the background areas show the range of emissions in the same month from 2011 to 2019.

Overall, the carbon footprints during the pandemic were within the range of the values from 2011 to 2019 without dramatic declines, consistent with the conclusions obtained in a previous study^44^. It can be inferred that the pandemic had some impact on city-level household carbon footprints for reasons related to travel restrictions and health concerns. Most months showed that total emissions were near the lower range of previous results, and some months had carbon footprints below that range. For example, during the study period, significant decreases were observed from January to April 2020 and from December 2020 to February 2021, while for months including June, September, October 2020, and March 2021, the total emissions rose to the mid-to-upper range of the pre-pandemic period.

By looking at each category of household demand, emissions from clothing and recreation decreased. Except for housing, the carbon footprints from residential demand in the ‘other’ categories did not change much compared with previous years, and their situations at different times need to be discussed separately. For instance, the carbon footprints of medical services were at a higher level compared to those of previous years, while education and home energy use were at lower levels; however, no major deviation was observed. To note, the changes in home energy consumption might be explained by the improved energy efficiency as well as the temperature differences between years rather than by COVID-19-related behavioral changes. Due to the pandemic, residents spent more time at home, resulting in higher footprints in the housing categories. Moreover, the footprints of transportation and communication were lower from April to May 2020, which was the period of the first stage of the emergency, and the footprints returned to the normal range in the following months. Contrary to expectation, CO_2_ emissions from home energy consumption did not increase significantly during the declaration of the state of emergency. This is because the weather in most areas of Japan is relatively mild in April and May, and the demand for heating and cooling is not high. However, CO_2_ emissions from home energy consumption in Sapporo, where heating demand is high even in April and May, increased significantly (see Fig. S7).

**Fig. 5:**
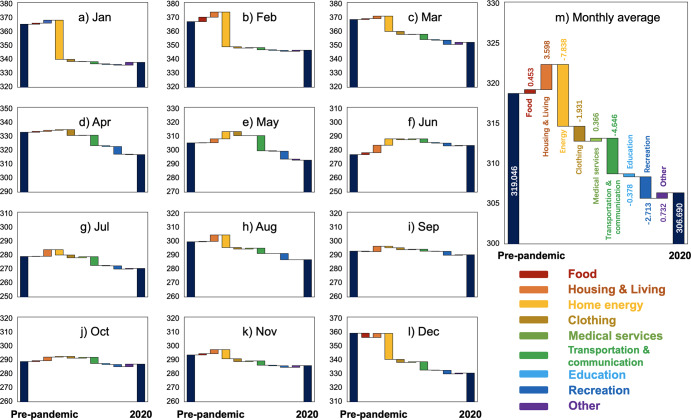
Total and monthly changes in carbon emissions (in kgCO_2_eq/cap/month) during the COVID-19 pandemic. **a**−**l** The results of individual months from January to December, and **m** The monthly average results.

To visualize the impact of each demand type on the carbon footprints of urban households, changes in carbon footprints for the whole year (Fig. 5m) and each month (Fig. 5a–i) in 2020 are compared to the average value of the corresponding month in previous years in the form of a waterfall chart. In this chart, the changes in carbon footprints in different periods are decomposed by demand type. At a holistic level, the average monthly emissions in 2020 are 0.013 tCO_2_eq/cap/month lower than those in previous years. This decrease can be attributed to a 0.008 tCO_2_eq/cap/month and a 0.005 tCO_2_eq/cap/month reduction in home energy use and transportation and communication demand, respectively, followed by a 0.003 tCO_2_eq/cap/month and a 0.002 tCO_2_eq/cap/month decline from the reduced demand for entertainment and clothing, respectively. Moreover, housing, the demand type that increased emissions the most, provide an increase of 0.004 tCO_2_eq/cap/month.

As for the monthly results, the carbon footprints are generally lower than the average values of the previous years for all months except June. According to the Office for COVID-19 and Other Emerging Infectious Disease Control, the first confirmed case in Japan was reported on January 26, 2020, and June 2020 was the month with the lowest number of confirmed cases after the beginning of the pandemic, with a rebound in July that peaked in August and a slowdown in September and October. For most months that showed a decrease in carbon footprints compared to previous years, although the extent of the decrease varied, the overall trend was similar. Moreover, although the reduction in emissions was related to the number of COVID-19 cases, the pandemic was not the only determining factor. For instance, between January–March and November–December, the demand for home energy use resulted in a significant decrease in footprints. With the onset of winter, the number of newly confirmed cases gradually increased, reaching another high in December; however, as mentioned above, the direct emission of Japanese households (the main energy consumers) showed a decreasing year-wise trend. Therefore, we cannot conclude that the carbon footprints from home energy use are affected only by pandemic factors.

Additionally, Supplementary Fig. 7 decomposes the emission gap before and during the pandemic by different household demand types and lists the results of four typical cities across Japan as examples. Sapporo and Naha were selected as they are the capital cities of the northeast Hokkaido and the southeast Okinawa prefectures, respectively. Two other cities, Tokyo and Osaka, are the economic centers of Japan. The emission gap decomposition indicates that the carbon footprint variances before and during the pandemic are impacted by both geographic (e.g., different weather conditions) and economic factors (e.g., Tokyo and Osaka have the highest population densities of all Japanese cities). In addition, the results of emission gap decomposition from Sapporo, Tokyo, Osaka, and Naha in four different months are given, and more information regarding other periods during the COVID-19 pandemic is also available from our results. One factor explaining the changes in the carbon footprint during these periods is the severity of the pandemic, and the number of daily confirmed cases in Japan is shown in Supplementary Fig. 8. More information on the COVID-19 situation and the measures promoted by the Japanese government with city-level carbon footprints in the household sector can be found in the Supplementary Information.

## Discussion

Considering that Japan is currently experiencing an accelerating depopulation and aging transition^45^, with more population and younger labor increasingly flowing into megacities^46,47^, the implementation of emission reduction policies would make limited progress if we relied solely on global and national calls without considering actions at the subnational level (considering the upgraded spatial heterogeneity). Due to the varying industrial activities and technological levels in different regions and the need to understand the spatial characteristics of carbon footprints better, increasing attention has been given to cities in recent years when conducting carbon accounting in Japan^18,48–50^. To date, Japan’s subnational carbon accounting has been conducted in certain defined aspects^49^, and the changes in household direct and indirect carbon emissions have been identified in a specific period^17,51^. For example, by the time September 30, 2022, 785 Japanese cities have participated in the ‘2050 Zero Carbon Cities in Japan’ announcement, indicating the recent progress of city insights^52^. Nevertheless, how to promote city-scale decarbonization pathways is still under discussion. Therefore, the current national decarbonization plans demand to be further downscaled to cities, with more city-scale emission information and city-specific customized measures available.

This study allows for helping policymakers fill these knowledge gaps and tackle the detailed emission abatements through the visualization of the commonalities and diversities of household carbon footprint across the cities in the 47 prefectures. As depicted in Fig. 2, there is a declining trend in the household carbon footprint from Hokkaido to Okinawa, and this is determined mainly by the degree of direct emissions. In this case, we observed a high dependence on kerosene for energy use in the northeast region of Japan from the perspective of direct emissions (see Supplementary Fig. 4). For this reason, the local government should set policy implementation goals by promoting renewable energy in cities and villages. For instance, subsidies for solar panels, home insulation, and energy-efficient machines would greatly benefit regions with lower winter temperatures.

The situation is quite differentiated for indirect emissions, and no major distinction is observed across regions (ranging from 0.21 tCO_2_ to 0.28 tCO_2_ across Japan). However, the proportion of indirect emissions is relatively higher than that of direct emissions, calling for policymakers to be mindful of the overall emissions of different consumption items from the perspective of their production and supply chains. For example, although the levels of direct carbon emissions are similar among all the seven cities except Naha in the Kyushu region (i.e., Naha is apart from Kyushu although it is included in the Kyushu-Okinawa area) as shown in Supplementary Fig. 4, the consumption items significantly contributing to the emission varied even in the same region (see Supplementary Fig. 5). For indirect emissions, Nagasaki and Kumamoto indicates low emissions from food consumption compared with the other cities, while Saga and Oita showed larger emissions from home energy use (mainly electricity) than the others. Besides, this study also provides the monthly variances of household carbon footprint among the cities, which could be utilized for a campaign for reductions in the specific consumption risen due to seasonal events (e.g., New Year Holidays during the end of December and head of January).

As pointed out by previous studies, the residential demand for goods and services differs by social class, economic status, and locations^53,54^, which is also well linked to the partial conclusion of this study. For example, households in metropolitan areas pay more attention to education, have greater access to medical care, and are willing to pay for entertainment^55,56^. Such preferences lead to larger carbon footprints for these categories in metropolitan areas from our findings (see Fig. 3). In addition, due to the better development of public transportation facilities, per capita emissions produced by transportation in large cities are lower than those in other cities (See Supplementary Fig. 3 and Supplementary Fig. 6). At the same time, the promotion of new energy vehicles in Tokyo is also one important reason behind its lower carbon footprint in transportation, given its goal to make the number of fast chargers reach 1000 by 2030, and the sales of new energy vehicles account for 50% of all vehicle sales, including electric vehicles, plug-in hybrid electric vehicles, and fuel cell vehicles. Hence, it is necessary to promote similar policies for areas with high emissions from transportation, such as Yamagata, Fukushima, Mito, Utsunomiya, and Maebashi, encouraging people to use environmentally friendly modes of transportation. Furthermore, we divide the emissions sources according to residents' needs, so that the emission responsibility is allocated to specific demand categories, thereby contributing to fine-grained policy recommendations.

Beyond the factors that may impact city-scale emissions considered in this study, understanding how demographic factors impact city-scale emissions, such as the aging society and the continued reduction in the effect of household size on the carbon footprint, is also important^17,57–59^. However, these factors still need to be discussed in future carbon footprint studies to determine how they affect urban household emissions. In addition, considering the observed trends of increasing affluence, consumerism, and population aging, Japan has become an ideal place to explore the environmental consequences brought about by lifestyle changes of different age groups, which are critical in providing information for regionally customized decarbonization plans.

Following the general analysis, this study also revealed the impact of COVID-19 on Japan’s household carbon footprints beyond the early diffusion period^44^ and generate the consistent conclusion that residents' behavior and lifestyle changes under the pandemic would not bring continual environmental benefits. Based on the emissions divided by household demands over 18 months, although people traveled less and spent more time at home due to COVID-19, no significant change in the household carbon footprint was observed in all relevant consumption categories. For Japanese households, the carbon footprint pattern decreases only slightly in categories such as clothing and recreation, which account for a rather small fraction of the household carbon footprint. Moreover, there are some categories, such as housing, food, and medical services, the carbon footprints of which are in the higher range but do not exceed the levels of previous years. The current finding indicates that a lifestyle of reduced activity during the unexpected social emergence period (such as COVID-19) did not result in a substantial household emission decrease. Despite the downward trend in the demand of the specified categories, the original consumption behaviors will eventually come back when the emergence situation goes mild, which cannot be recognized as a sustainable decarbonization measure. However, we also acknowledge that if the social emergency lasted for years and has changed the production ways, the impact will finally reach the consumption terminal and result in a sustainable change.

Based on the data analysis so far, our conclusion generated from the consumption perspective shows a different picture from early conclusions from the production side. For example, it has been confirmed that lockdown measures to prevent the spread of COVID-19 have resulted in decreased mobility trends^60,61^, a significant drop in air pollution (NO_*x*_, PM_2.5_, SO_2_, etc.) and CO_2_ emissions through atmospheric observation^62–64^. However, temporary constraints on the movement of residents, such as limiting public transportation and suggesting home offices, cannot lead to sustainable environmental relief. Since the consumption structure did not change during the pandemic, there exists the risk of rebound effects after these restrictions are lifted^65^. In this case, the disruption of domestic production, supply, and international trade was not intentional, but its impact reflected the possible changes in the current carbon footprint (e.g., higher household consumption or reduced recreational activities) caused by pandemic preparedness measures. In addition, previous social and economic crises suggest that such carbon footprint reductions are short-lived, and emission levels might rebound and return to their original level. For example, during the 2008 global financial crisis, Japan’s CO_2_ emissions fell in 2009 but increased in 2010 to follow the previous trajectory, as if the crisis had not occurred^66^. For COVID-19, such a temporary downturn and stagnation in emissions from production would be reversed by demand stimulation. Furthermore, if the pandemic continues and we step into a ‘with-COVID-19 era’ (co-existed with COVID-19), we may see similar results as a financial crisis or earthquake as shown before. Therefore, whether the short-term pandemic could result in consistent mitigation is questionable.

In conclusion, numerous means to stimulate economic recovery have been proposed, and investment choices for economic recovery will strongly influence the course of global warming^67,68^. According to the current analysis, reducing the carbon footprint still depends on technological advances, particularly in the development of cleaner energy. Without a long-term, systematic decarbonization economy, any behavioral shifts will lead to only a modest reduction in the rate of global warming. However, it is also essential to note that this study has limitations. Currently, the focus is limited to the urban level and only examines eight prevalent categories of household spending. While over 500 household consumption items have been quantified in terms of their implicit carbon footprint, this simplified classification was deemed necessary to offer a more straightforward understanding due to the substantial amount of data. In the future, research will expand the examination of household carbon footprint to include a wider array of perspectives, with an aim to inform the development of environmentally friendly economic stimulus policies that take into account regional/city-level variations, in support of sustainable recovery from the impacts of COVID-19 and in line with long-term climate goals.

## Methods

### Calculation of direct carbon emissions from households

The fossil fuels that lead to direct household emissions are gasoline, kerosene, liquefied petroleum gas (LPG), and city gas. A weekly survey on retail prices at filling stations^69^ conducted by the Ministry of Economy, Trade, and Industry of Japan provides retail prices for gasoline and kerosene each week. Monthly prices for gasoline and kerosene are then calculated as the mean value of weekly prices for each month. For kerosene, the prices for on-site purchases are adopted because a survey on kerosene and LPG purchases shows that most Japanese families purchase fuel in this way^70^. The prices of LPG in the 47 prefectural-level cities are from the Oil Information Center at the Institute of Energy Economics^71^. As LPG retail prices are documented by volume step-by-step (at 5, 10, 20, and 50 m^3^), the prices of LPG in cities are also stepwise by volume and are slightly above the mean value of monthly purchase volume. The average volume purchased per month in each prefecture is provided by updated data on LPG consumption in the 47 prefectural-level cities^72^. To demonstrate how the price data are obtained, Osaka is taken as an example. Here, the average monthly consumption of LPG is 13.8 m^3^ per household, and the price of 20 m^3^ LPG (JPY/m^3^) is adopted to reflect the price per unit LPG, as 20 m^3^ is the stepwise volume that is just above 13.8 m^3^. Based on Japanese government statistics, we have gathered the city gas prices from 2011 to 2014^73^. Due to the lack of information on city gas prices since 2015, Japan’s Consumer Price Index has been applied to transform data from 2014 for the following years^74^.

The per capita direct emissions can then be calculated from household expenditures using the following equation:

1 $$E_{j,t,m}^{r,{{{\mathrm{direct}}}}} = f_{j,k,t}^{{{{\mathrm{direct}}}}}y_{j,t,m}^{r,{{{\mathrm{FIES}}}}}/h_t^{r,{{{\mathrm{FIES}}}}}$$

where $E_{j,t,m}^{r,{{{\mathrm{direct}}}}}$ is the per capita direct emission of expenditure item *j* in city *r* month *m* year *t*, $f_{j,k,t}^{{{{\mathrm{direct}}}}}$ the direct emission intensity of expenditure of item *j* and fuel type *k* in year *t*; and $y_{j,t,m}^{r,{{{\mathrm{FIES}}}}}$is the expenditure on item *j* and city *r* of month *m* year *t* from the Family Income and Expenditure Survey (FIES). To note, to calculate $y_{j,t,m}^r$ the monthly price fluctuation is also considered as mentioned above. The direct emission intensity of expenditures $f_{j,k,t}^{{{{\mathrm{direct}}}}}ss$ is calculated using the emission coefficients of gasoline, kerosene, LPG, and city gas, which are provided by Japan’s Ministry of the Environment^75^. The emission coefficient of each fuel, which describes the quantity of CO_2_ emissions corresponding to unit mass or volume, is first converted into the CO_2_ intensity of expenditure (i.e., the quantity of CO_2_ emissions per unit expenditure, in g-CO_2_-eq/JPY) through the retail prices of fuels. The LPG’s emission coefficient is organized with tCO_2_/ton as the unit, as presented in the dataset, and its retail prices adopt the unit of JPY/m^3^, which is achieved through the weight of liquid LPG (in tons) transformed into the volume of gaseous LPG (in m^3^) by the conversion coefficient advised by the Ministry of the Environment^76^. $h_t^{r,{{{\mathrm{FIES}}}}}$ indicates the average household size by city *r* in year *t* captured from FIES.

### Calculation of indirect carbon emissions of households

To quantify the indirect emissions embodied in the goods and services that satisfy the household sector, cross-mapping using the input–output lifecycle inventory dataset (3EID^26,77^) and the FIES dataset^78^ is required, and the GHGs (CO_2_, CH_4_, N_2_O, HFCS, PFCS, SF_6_, and NF_3_) are included in the calculations. The FIES survey selected approximately 9000 households from appropriate households (excluding households such as single student occupants, hospital inpatients, foreign households, etc.). In order to avoid bias in the obtained numbers and to free the sampled households from the burden of long-term bookkeeping, the sample was updated periodically. The calculation of the indirect carbon emission intensity $( {f_{j,t}^{{{{\mathrm{indirect}}}}}})$ in the 3EID is modeled as follows:

2 $$\left( {\begin{array}{*{20}{c}} {f_{1,t}^{{{{\mathrm{indirect}}}}}} \\ \vdots \\ {f_{j,t}^{{{{\mathrm{indirect}}}}}} \\ \vdots \\ {f_{n,t}^{{{{\mathrm{indirect}}}}}} \end{array}} \right) = {{{\mathbf{D}}}}({{{\mathbf{I}}}} - ({{{\mathbf{I}}}} - {{{\bar{\mathbf M}}}}){{{\mathbf{A}}}})^{ - 1}$$

where ${{{\mathbf{D}}}} = \left[ {d_i} \right]$ denotes the direct emission intensity vector, **I** represents the unit matrix, ${{{\mathbf{A}}}} = [ {A_{ij}}] = [ {\frac{{x_{ij}}}{{X_j}}}]$, where *x*_*ij*_ is industry *i*’s output needed to produce industry *j*’s output, *x*_*j*_ is the total output of sector *j*, and ${{{\bar{\mathbf M}}}}$ is the diagonal matrix symbolizing the direct requirement coefficients for the import portion. Due to structural limitations, the 3EID considers only domestic production, and the specific details of the input–output table and applications are available in other studies^11,19,25,79–83^.

Due to the differences between the 3EID database’s industry classifications and consumption elements in the FIES expenditure data, we rematched the data according to a method described elsewhere^18^ and sketched out a feasible approach for acquiring data for the year 2015 in one of the supporting documents. A total of 395 items were included in the emission intensity dataset 3EID in 2011 and 390 items in 2015. Based on the results obtained through cross-mapping the 3EID datasets with the corresponding FIES datasets, we obtained an emission inventory with 495 items between 2011 and 2014, 512 items between 2015 and 2019, and 504 items between 2020 and 2021.

Although the data regarding household expenditures on goods and services falling under indirect emissions were provided by the FIES on a monthly basis between January 2011 and June 2021, the indirect emission intensities relevant for each of these indirect emission categories after cross-mapping (see above) were generated only for the 2011 and 2015 input–output tables. This is because the 3EID databases used for the emission intensities are released every five years, and the closest ones related to our data are from 2011 and 2015^84^. Regarding the above reasons, we took the interpolation method with inflation and CPI to bridge the input–output table with missing years to investigate the carbon footprint of Japanese urban households over a relatively wide time span. This is motivated by several global input–output databases that extend MRIO tables to the most recent years by correlating them with currency and GDP. Nevertheless, we must acknowledge that the results obtained using interpolation contain uncertainty, especially considering the changes in the population’s consumption patterns since the outbreak of COVID-19. Thus, linear interpolation is applied to assess the indirect emission intensities for all study items for the relevant years, thereby obtaining the values. The expressions related to the interpolation method are as follows:

3 $$\left\{ {\begin{array}{*{20}{c}} {f_{j,2012}^{{{{\mathrm{indirect}}}}} = \frac{3}{4}f_{j,2011}^{{{{\mathrm{indiret}}}}} + \frac{1}{4}f_{j,2015}^{{{{\mathrm{indirect}}}}}} \\ {f_{j,2013}^{{{{\mathrm{indirect}}}}} = \frac{1}{2}f_{j,2011}^{{{{\mathrm{indirect}}}}} + \frac{1}{2}f_{j,2015}^{{{{\mathrm{indirect}}}}}} \\ {f_{j,2014}^{{{{\mathrm{indirect}}}}} = \frac{1}{4}f_{j,2011}^{{{{\mathrm{indirect}}}}} + \frac{3}{4}f_{j,2015}^{{{{\mathrm{indirect}}}}}} \\ {f_{j,2016}^{{{{\mathrm{indirect}}}}} = INF_{j,2016} \ast f_{j,2015}^{{{{\mathrm{indirect}}}}}} \\ {f_{j,2017}^{{{{\mathrm{indirect}}}}} = INF_{j,2017} \ast f_{j,2015}^{{{{\mathrm{indirect}}}}}} \\ {f_{j,2018}^{{{{\mathrm{indirect}}}}} = INF_{j,2018} \ast f_{j,2015}^{{{{\mathrm{indirect}}}}}} \\ {f_{j,2019}^{{{{\mathrm{indirect}}}}} = INF_{j,2019} \ast f_{j,2015}^{{{{\mathrm{indirect}}}}}} \\ {f_{j,2020}^{{{{\mathrm{indirect}}}}} = INF_{j,2020} \ast f_{j,2015}^{{{{\mathrm{indirect}}}}}} \\ {f_{j,2021}^{{{{\mathrm{indirect}}}}} = INF_{j,2021} \ast f_{j,2015}^{{{{\mathrm{indirect}}}}}} \end{array}} \right.$$

where $f_{j,t}^{{{{\mathrm{indirect}}}}}$ indicates the embodied carbon emission intensity of item *j* in year *t*. $f_{j,2011}^{{{{\mathrm{indirect}}}}}$ and $f_{j,2015}^{{{{\mathrm{indirect}}}}}$ are generated from 3EID^26,77^, which applied the 2011 and 2015 Japan input–output tables, respectively. From 2016 to 2021, the emission intensity was adjusted based on the 2015 emission intensity, with a modification of inflation ($INF_{j,t}$) of item *j* in year *t* (*t*: 2016, 2017, 2018, 2019, 2020, and 2021), which is driven by the Economic and Social Research Institute, Cabinet Office of Japan^85^. Notably, electricity emissions are also included in indirect emissions because emissions are not emitted while electricity is in use. Although we focus on multiple cities in Japan, the indirect emission intensity is unified across Japan.

The per capita indirect emissions can then be calculated from household expenditures using the following equation:

4 $$E_{j,t,m}^{r,{{{\mathrm{indirect}}}}} = \frac{{f_{j,t}^{{{{\mathrm{indirect}}}}}y_{j,t,m}^{r,{{{\mathrm{FIES}}}}}}}{{h_t^{r,{{{\mathrm{FIES}}}}}}}$$

Here, $E_{j,t,m}^{r,{{{\mathrm{indirect}}}}}$ indicates the per capita indirect emission of item *j* in year *t* month *m* in city *r*. $y_{j,t,m}^{r,{{{\mathrm{FIES}}}}}$ is the monetary consumption of item *j* in year *t* month *m* in city *r* from FIES.

### IDW interpolation of the emission distribution in Japan

Tobler’s first law of geography states that all attribute values on a geographic surface are related, but closer values are more strongly related than distant values^86^. The spatial distribution of carbon emissions reflects the economic activity and energy consumption in different areas and should be combined to guide the formulation of emission reduction guidelines and policies in different regions to address climate change issues^87^. For better visualization of the household direct/indirect carbon footprint distribution across Japan, IDW interpolation was used to generate distribution trends based on the information provided by major cities. The calculation process was as follows:

5 $$d_r = \sqrt {(x_0 - x_r)^2 + (y_0 - y_r)^2}$$

6 $$\lambda _r = \frac{{d_i^{ - p}}}{{\mathop {\sum }\nolimits_{r = 1}^n d_r^{ - p}}}$$

7 $$\hat P\left( {x_0,y_0} \right) = \mathop {\sum}\limits_{r = 1}^n {\lambda _rP(x_r,y_r)}$$

where *d*_*r*_ is the distance between two points, *λ*_*r*_ is the weighting term, $\hat P\left( {x_0,y_0} \right)$ is the interpolation value, *n* is the number of available data points near $\hat P\left( {x_0,y_0} \right)$, and *x* and *y* Arepresent the geographical locations of cities *r* and *s*. −*p* is an arithmetic number. Using the above equations, we can obtain a direct understanding of the carbon footprint by source. The geological data processing platform used in this study was ArcGIS 10.4.

### Reporting summary

Further information on research design is available in the Nature Research Reporting Summary linked to this article.

## Supplementary information

Supplemental Material

Reporting Summary

## Data Availability

All data aggregated or analyzed in the current study are available from the corresponding author on reasonable request. In this research, weekly retail prices for fuels are from the Ministry of Economy, Trade, and Industry of Japan (https://www.enecho.meti.go.jp/statistics/petroleum_and_lpgas/pl007/results.html), the Agency of Natural Resource and Energy (https://www.enecho.meti.go.jp/en/category/whitepaper/), and Oil Information Center at the Institute of Energy Economics (https://oil-info.ieej.or.jp/price/price.html.). Detailed information on household expenditures of Japan is from FIES (https://www.stat.go.jp/english/data/kakei/index.html). Emission intensity data are from Japan’s Ministry of the Environment (https://ghg-santeikohyo.env.go.jp/calc) and Economic and Social Research Institute, Cabinet Office of Japan (https://www.esri.cao.go.jp/en/sna/menu.html).

## Code availability

Upon reasonable requests, the corresponding authors can provide access to the code that was aggregated and/or analyzed during the current study.
