# Supplementary material for: Japanese urban household carbon footprints during early-stage COVID-19 pandemic were consistent with those over the past decade
Source: NPJ Urban Sustain. 2023 Mar 29;3(1):19. doi: 10.1038/s42949-023-00095-z (PMC10052282; doi:10.1038/s42949-023-00095-z)
Supplement: Supplementary file 1 — Supplemental Material [file 42949_2023_95_MOESM1_ESM.docx]

**Supplementary information**

*for*

**Japanese urban household carbon footprints during early-stage COVID-19 pandemic were consistent with those over the past decade**


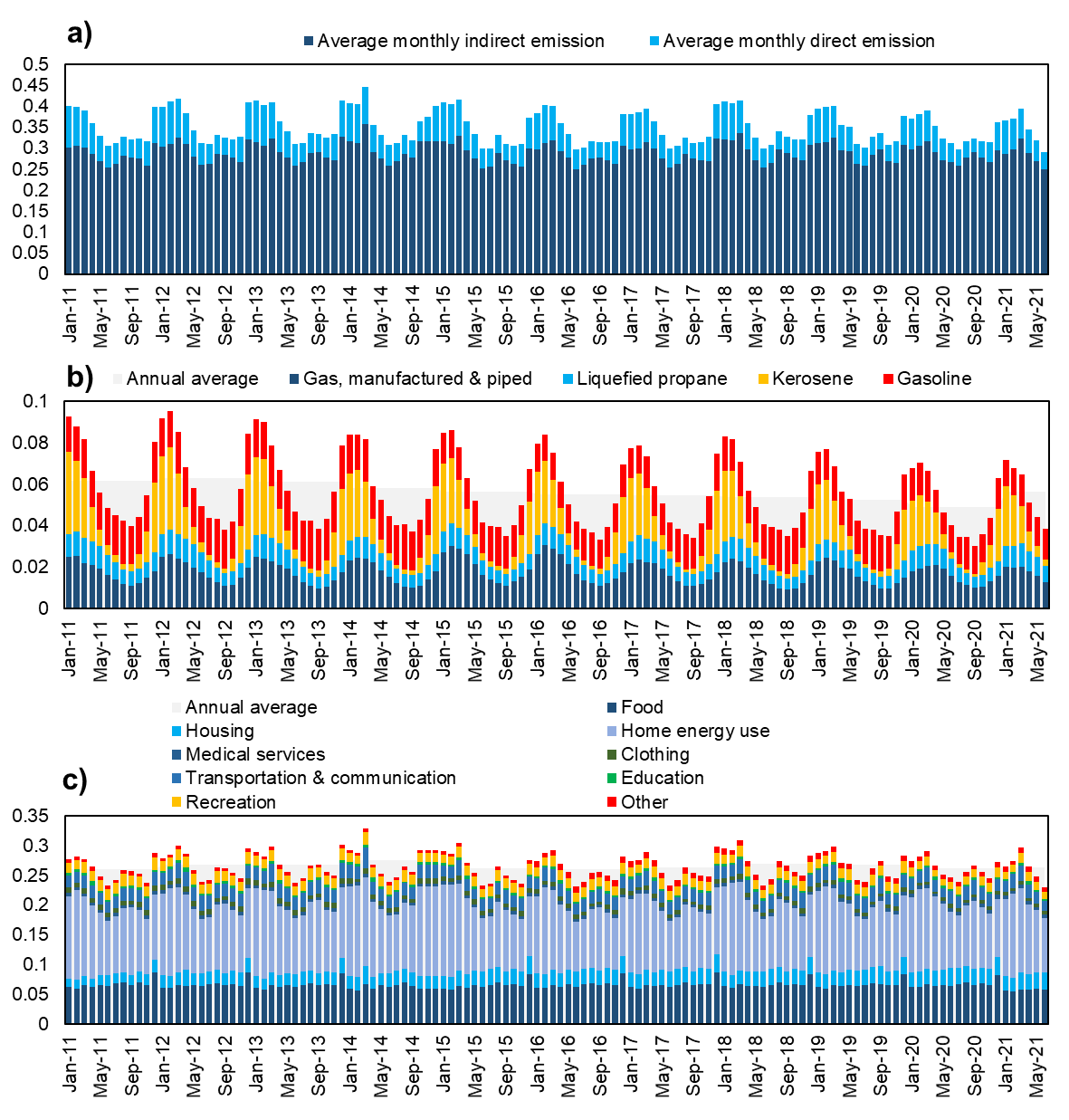


**Supplementary Figure 1. Household carbon footprints by month (in tCO_2_eq/cap/month) from 2011 to June 2021. Panel (a) total carbon footprint by direct and indirect emission; (b) direct emission by energy type, and (c) indirect emission by expenditure type.**

For direct emissions, pronounced surges can be observed from the fourth quarter to the first quarter of the following year, partly due to the increased kerosene consumption. Generally, power generation requires more petroleum products during the winter season, and the demand for heating increases as the temperature drops ^1,2^, such that direct emissions show a seasonal pattern. Emissions from natural gas show a similar pattern but the intensity of variation was lesser than that of kerosene emissions, and the gasoline and LPG-induced emissions do not change much throughout the year.

In comparison, the seasonal pattern of variation is less prominent for indirect emissions due to the integral impact of all expenditure types, which are typically larger between December and the following April and lower in the remaining months. Regarding the expenditure categories, a noteworthy point is that consumption in the food category causes indirect emissions to be highest in December for all years (except for 2014). Due to the seasonality of the indirect emissions of these two categories, the overall indirect emissions show a corresponding pattern, and other expenditure categories do not have observable seasonality. The total emissions were determined by direct and indirect emissions and showed the same trend.


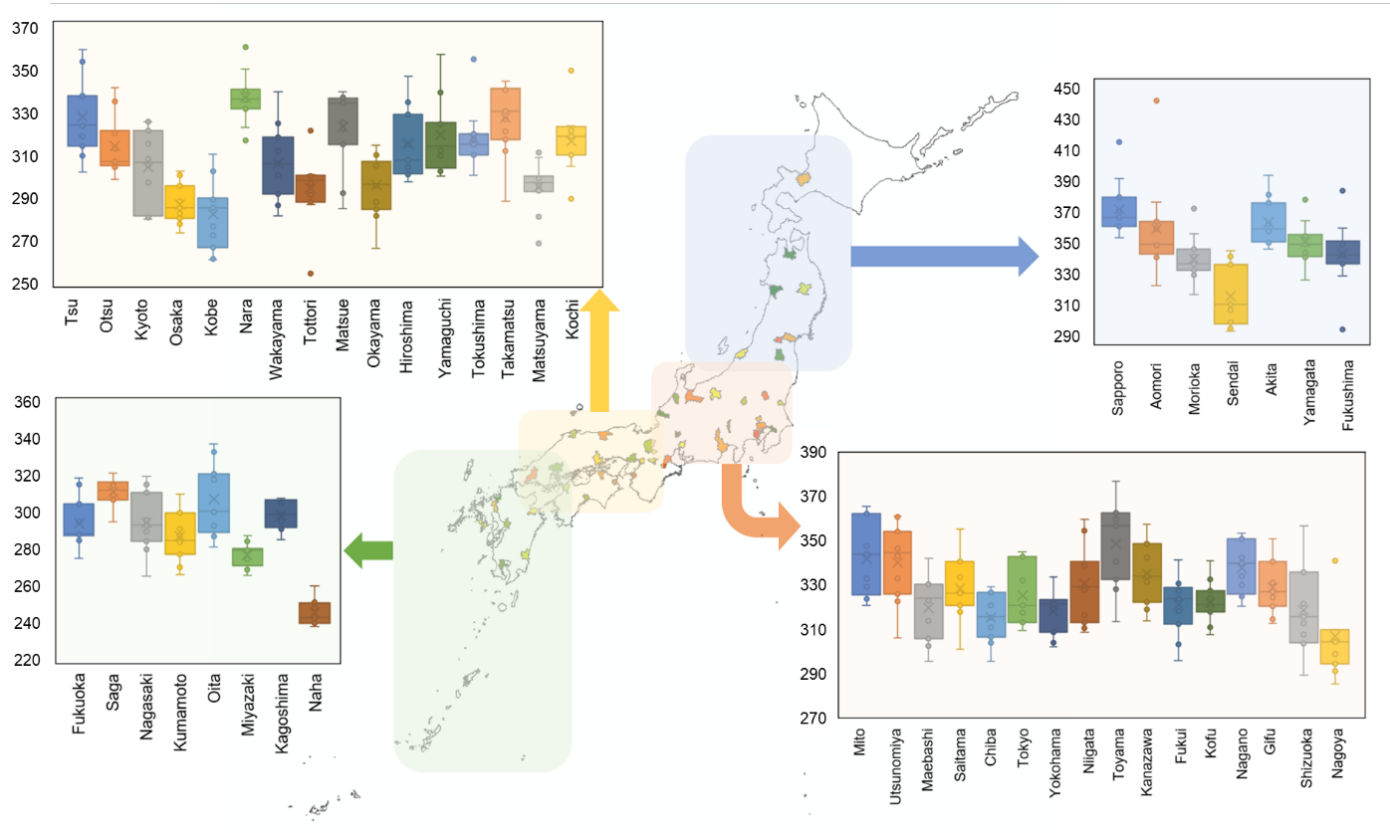


**Supplementary Figure 2. Distribution of monthly total carbon footprints of 47 Japanese cities from 2011 to June 2021 (kgCO_2_eq/cap/month).**

Such a mode of representation is beneficial for reflecting the emission distribution of each city and allows comparison among them. In addition, anomalous values in the data are worthy of attention, and the boxplot identifies anomalous footprint values. For example, for Kochi and Matsuyama, there were significantly small values in 2020, which would be due to the impact of COVID-19, and the monthly average emission results for the first half of 2021 are easily detected as large anomalous values in the figure. Including outliers in the data analysis can have an adverse effect on the results and lead to inaccurate measurement of emissions. The distribution results show that the cities with the highest annual monthly carbon footprints are Sapporo and Akita (0.371 tCO_2_eq/cap and 0.366 tCO_2_eq/cap, respectively), and that with the lowest is Naha (0.246 tCO_2_eq/cap). The spatial distribution is consistent with the aforementioned trend of a gradual decrease from north to south.


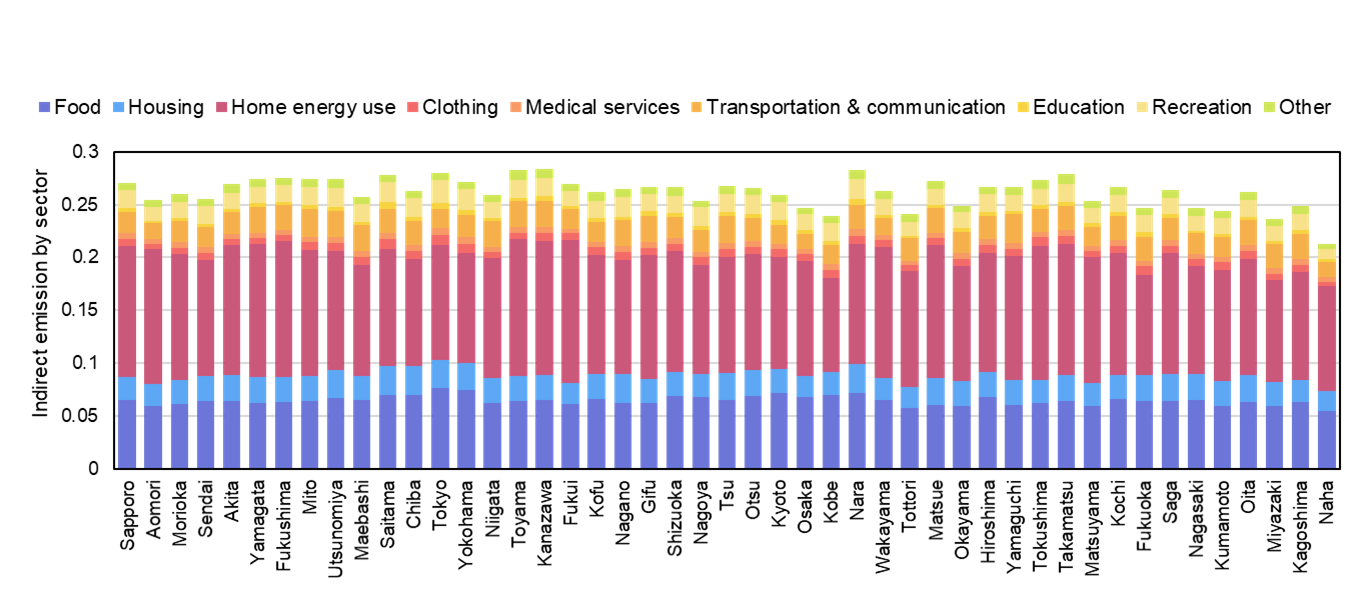


**Supplementary Figure 3. Monthly indirect carbon emissions by sector (tCO_2_eq/cap/month) in 47 cities of Japan from 2011 to 2021.**


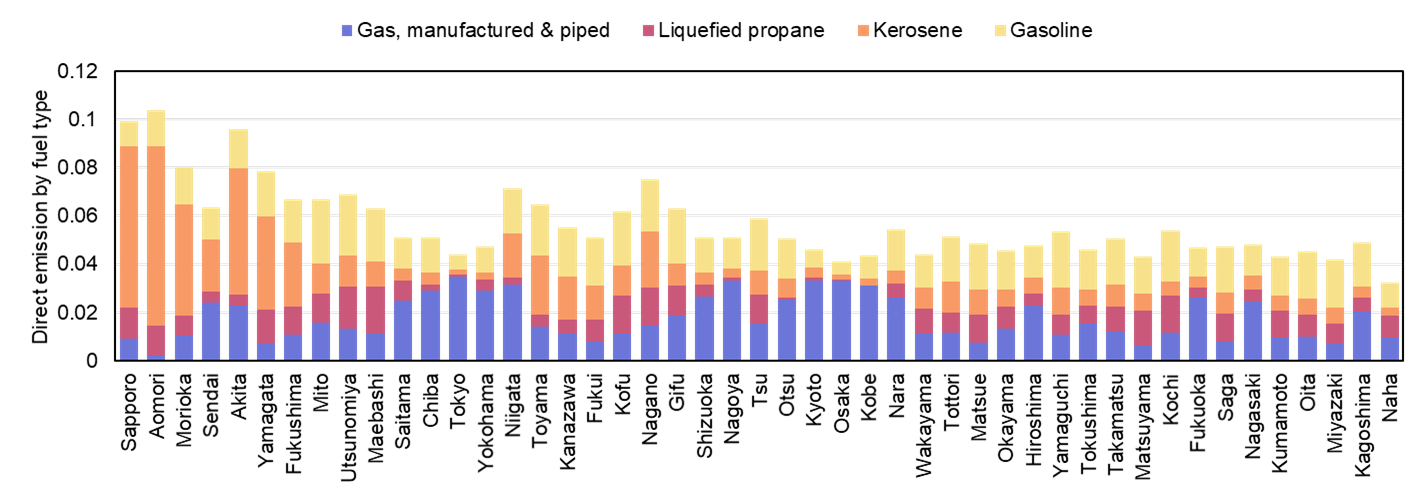


**Supplementary Figure 4. Monthly direct carbon emissions by fuel type (tCO_2_eq/cap/month) in 47 cities of Japan from 2011 to 2021.**


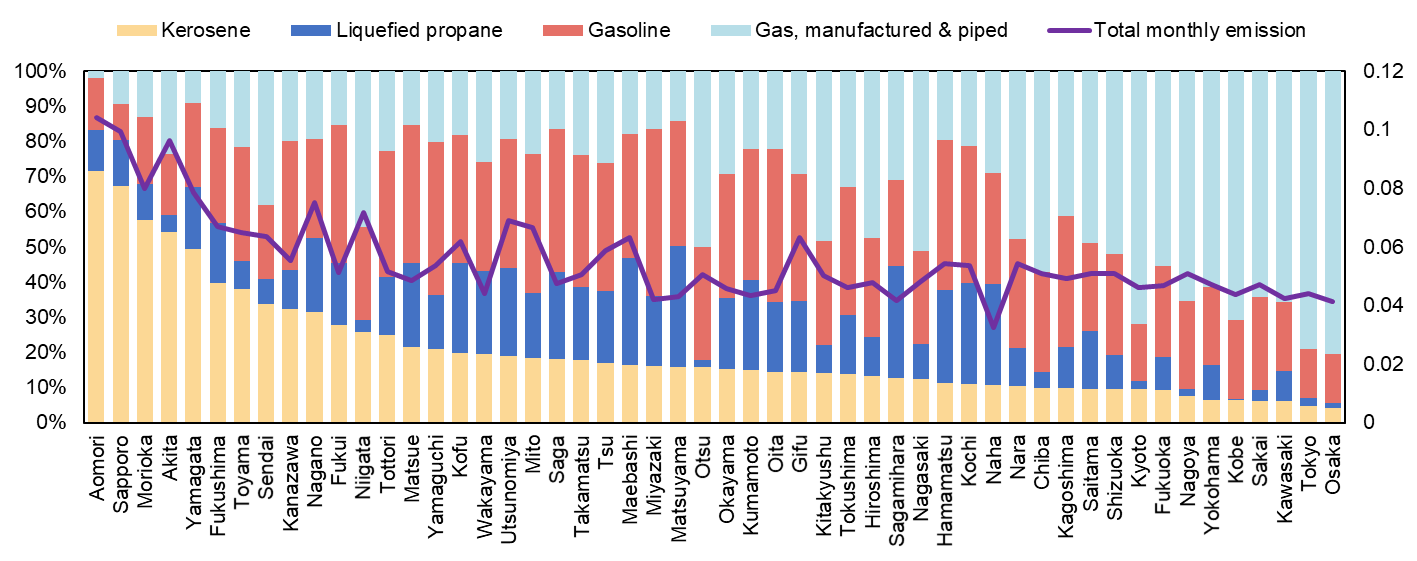


**Supplementary Figure 5. Composition of direct emission by fuel type (tCO_2_eq/cap/month) in 51 Japanese cities.**


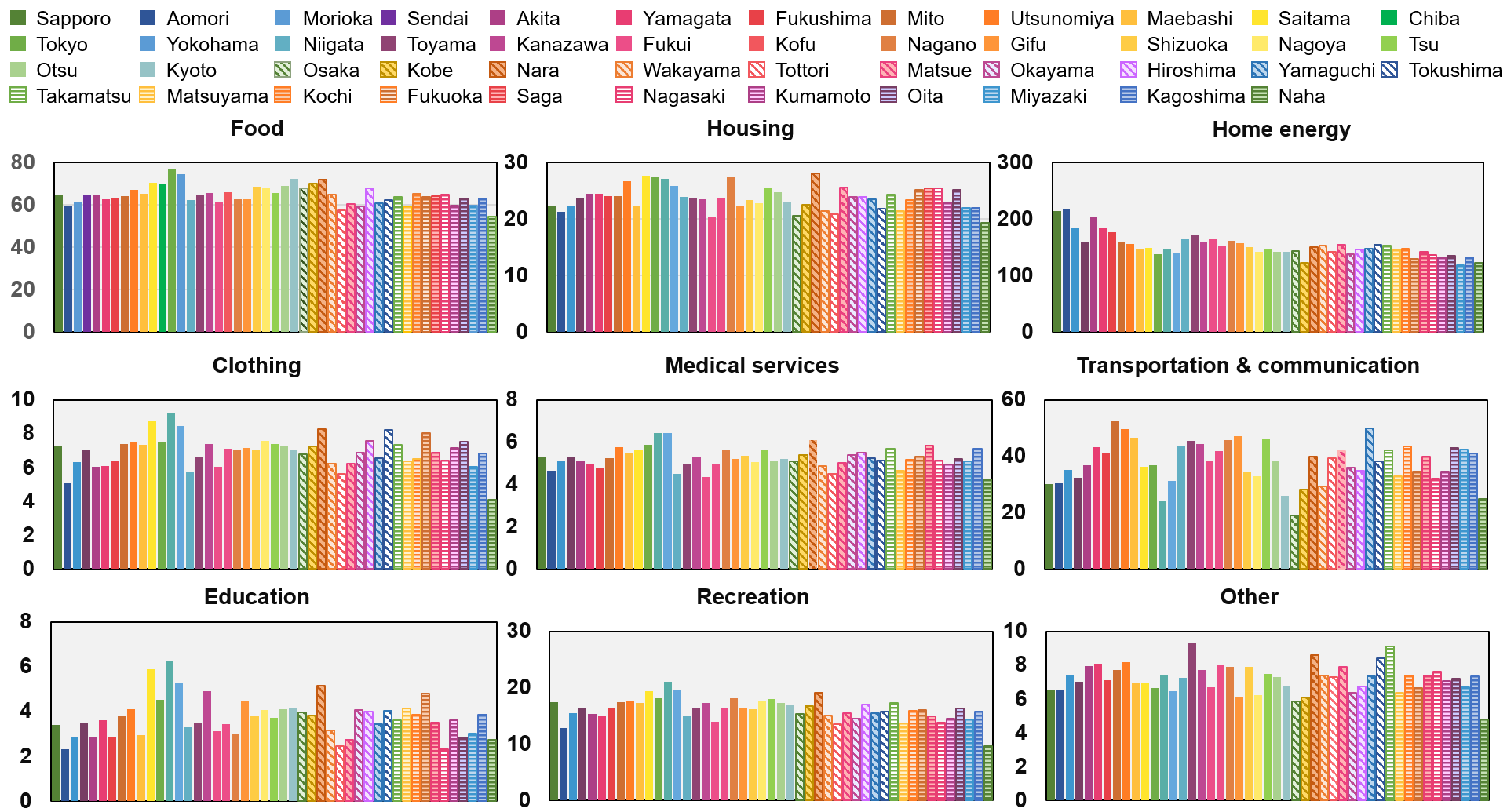


**Supplementary Figure 6. Results of carbon footprints by demand (kgCO_2_eq/cap/month) in 47 Japanese cities.**


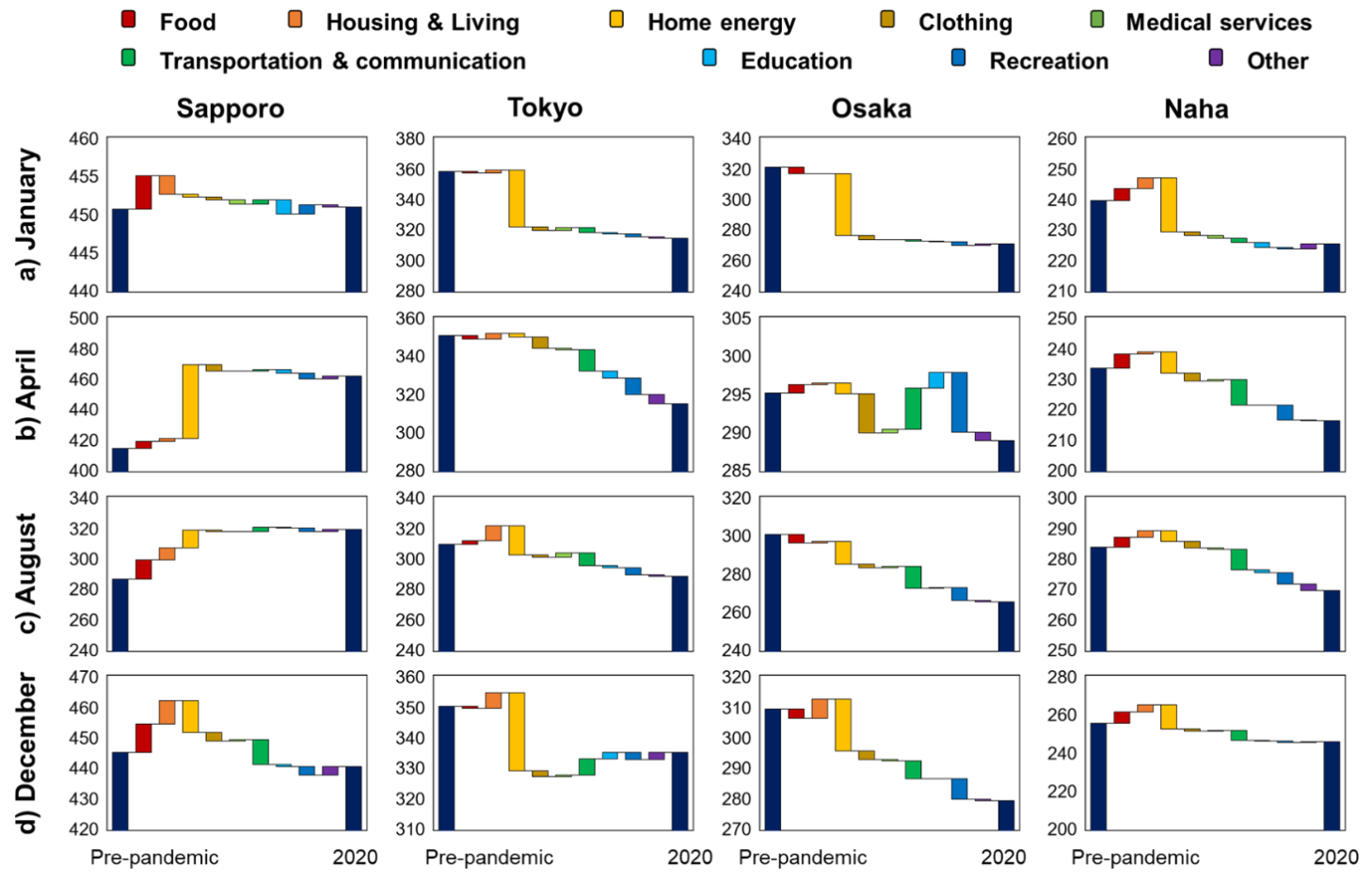


**Supplementary Figure 7. Comparison of carbon footprints by demand category in four typical Japanese cities during the pandemic (kgCO_2_eq/cap/month)**

We present the results of household carbon footprints in January, April, August, and December 2020, and the pre-pandemic values referenced are the historical averages of the corresponding cities for that month from 2011 to 2019. As noted in the figure, the impact of the pandemic varied considerably in different cities, but the differences among cities were obvious. For instance, Tokyo and Osaka witnessed larger carbon emission reductions in general than the other two cities. In addition, the household carbon footprints in Sapporo rose in April due to the increased home energy consumption. Although people spend more time at home during this period and cut down their outdoor activities and transportation demands, other home consumption needs, such as heating, have brought about changes in emissions due to household energy use because Hokkaido is relatively cold compared to the other three cities ^3^. Overall, the changes in city-level household carbon footprints during the pandemic were not significant; even with a short time increase, the levels later returned to normal (e.g., April 2020, in Sapporo), and the household decarbonization pathway was not deeply impacted. This implies that COVID-19 prevention measures are not likely to be effective in reducing carbon emissions. Therefore, to reduce carbon emissions, end-use reduction policies, such as electrification are required.

**
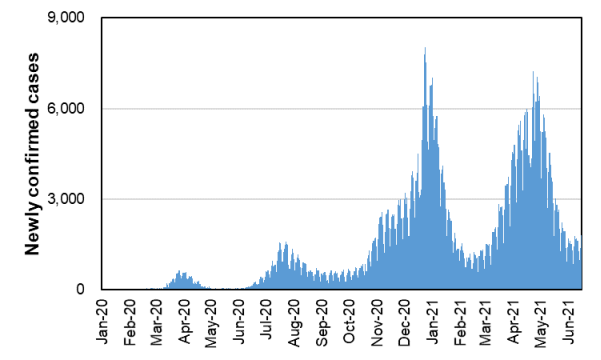
**

**Supplementary Figure 8. Trend in the number of newly confirmed cases in Japan (daily).**

For months at the beginning of the COVID-19 pandemic, emission reductions were relatively significant. In April 2020, the first state of emergency was announced in Tokyo and six other prefectures, which was then extended to the entire country. However, this was not a lockdown, as public transportation remained operational ^4^. Despite adopting more lenient measures than other countries, Japan reported its lowest number of daily cases of COVID-19 from April 20 to June 10, 2020. The first state of the emergency was completely lifted on May 25, 2020. The carbon footprints from May to November were within the levels of the previous years. Aiming to support tourism, on July 22, the government launched a 50% subsidized travel program named the "Go To Travel Campaign" (GTTC), with government-issued coupons for tourist attractions ^5^, which partly accounts for the footprint levels from July to November. The pandemic situation gradually worsened after November 2020, and the number of confirmed cases peaked at the end of December, which may help explain the apparent decrease in the carbon footprint from December 2020 to February 2021. Residents in the affected areas were asked to refrain from all nonessential excursions. All drinking and dining establishments were asked to close at 8 p.m. (local time). As the number of infected people in the country exceeded 300,000, the second state of emergency in Tokyo and three neighboring prefectures was declared on January 7, 2021, and eight other prefectures, including Osaka, Kyoto, and Fukuoka, were then placed under the state of emergency ^6,7^. Although not all cities in Japan were included in this state of emergency, residents in the affected areas were asked to refrain from all unnecessary excursions, and the opening hours of many establishments were restricted^8^.

The second state of emergency ended on March 7, 2021, in six prefectures and was lifted on March 22 in Tokyo and three neighboring prefectures. By February 2021, the number of confirmed cases declined, and another wave did not emerge until April, indicating that the relatively high footprints in March were due to fewer confirmed cases and the lifting of the state of emergency. However, a sudden increase in the number of confirmed cases occurred, and the third state of emergency was declared on April 23, 2021, for Tokyo and the western prefectures of Osaka, Kyoto, and Hyogo ^9^. Unlike the initial pandemic stage (January-April 2020) and the previous wave (December 2020–February 2021), the third state of emergency and priority preventative measures issued were maintained for a relatively long period and were not lifted in all prefectures until September 30, 2021. Even though the number of confirmed cases was high and relevant policies were in place to constrain people's activities, no significant reduction in emissions was observed during this period. This may be because residents had become accustomed to basic infection prevention measures (such as avoiding "the Three Cs": closed spaces with poor ventilation, crowded places with many people nearby, and close-contact settings with as close-range conversation), and the successful development and administration of vaccines had created the possibility of restoring daily life. In summary, the above results indicate that many of the impacts of COVID-19 on the carbon footprint will not be long-term.

**Supplementary Table 1. Average monthly GHG emissions of 47 prefectural-level cities before the pandemic (from 2011 to 2019, kgCO_2_eq/cap/month)**

|  | January | February | March | April | May | June | July | August | September | October | November | December |
| --- | --- | --- | --- | --- | --- | --- | --- | --- | --- | --- | --- | --- |
| Sapporo | 450.7 | 459.7 | 460.6 | 414.1 | 364.7 | 301.1 | 276.8 | 286.7 | 273.9 | 305.0 | 338.9 | 445.0 |
| Aomori | 454.6 | 443.5 | 435.7 | 384.2 | 326.7 | 276.9 | 264.6 | 274.4 | 275.8 | 297.3 | 333.9 | 440.9 |
| Morioka | 430.9 | 426.5 | 427.8 | 372.2 | 325.9 | 290.4 | 272.8 | 274.3 | 257.3 | 281.7 | 305.2 | 401.4 |
| Sendai | 376.8 | 369.9 | 364.8 | 338.2 | 312.3 | 277.7 | 279.4 | 285.8 | 261.5 | 277.2 | 305.9 | 363.2 |
| Akita | 446.8 | 441.6 | 435.0 | 384.2 | 335.8 | 294.4 | 285.4 | 294.4 | 288.5 | 312.1 | 350.1 | 439.5 |
| Yamagata | 427.1 | 438.6 | 432.7 | 368.2 | 334.4 | 287.2 | 280.8 | 286.6 | 284.1 | 300.3 | 321.8 | 432.0 |
| Fukushima | 420.5 | 405.5 | 399.0 | 342.6 | 320.4 | 291.9 | 295.5 | 308.4 | 286.7 | 300.4 | 329.5 | 413.3 |
| Mito | 394.4 | 392.7 | 393.2 | 357.5 | 325.5 | 302.6 | 299.4 | 323.0 | 325.2 | 311.3 | 320.1 | 396.1 |
| Utsunomiya | 391.0 | 393.1 | 409.8 | 358.8 | 342.4 | 307.3 | 307.4 | 315.6 | 310.6 | 312.8 | 317.3 | 384.9 |
| Maebashi | 367.8 | 365.9 | 370.6 | 327.8 | 307.9 | 280.3 | 271.8 | 299.4 | 306.4 | 294.8 | 302.2 | 357.9 |
| Saitama | 361.6 | 372.9 | 392.8 | 354.1 | 322.1 | 287.8 | 304.0 | 310.6 | 308.4 | 297.0 | 302.7 | 352.2 |
| Chiba | 362.9 | 365.7 | 365.1 | 343.3 | 302.9 | 267.9 | 269.2 | 291.2 | 292.3 | 283.2 | 292.5 | 351.4 |
| Tokyo | 360.0 | 367.2 | 375.5 | 351.3 | 320.5 | 287.9 | 295.4 | 310.3 | 316.1 | 298.9 | 294.0 | 350.4 |
| Yokohama | 349.0 | 361.4 | 357.6 | 343.7 | 311.4 | 280.2 | 290.6 | 311.8 | 318.4 | 284.5 | 299.1 | 348.1 |
| Niigata | 386.0 | 384.0 | 383.8 | 346.3 | 327.5 | 285.3 | 271.5 | 295.8 | 281.0 | 303.0 | 320.9 | 374.4 |
| Toyama | 390.8 | 413.3 | 419.6 | 382.7 | 323.2 | 304.8 | 293.5 | 315.8 | 310.8 | 303.9 | 318.8 | 389.9 |
| Kanazawa | 381.6 | 399.4 | 399.4 | 354.1 | 320.0 | 295.2 | 292.5 | 314.1 | 295.7 | 308.3 | 321.3 | 390.0 |
| Fukui | 342.0 | 370.4 | 371.1 | 317.7 | 305.8 | 284.6 | 277.0 | 307.6 | 298.8 | 300.3 | 285.4 | 354.4 |
| Kofu | 365.1 | 382.0 | 371.8 | 329.2 | 309.8 | 276.9 | 281.4 | 302.0 | 311.1 | 296.2 | 292.7 | 378.7 |
| Nagano | 408.7 | 401.2 | 401.5 | 356.5 | 327.5 | 289.7 | 281.1 | 296.2 | 295.4 | 292.9 | 331.8 | 398.4 |
| Gifu | 369.6 | 359.4 | 372.3 | 336.8 | 316.4 | 288.2 | 298.1 | 313.8 | 305.7 | 295.4 | 305.8 | 367.5 |
| Shizuoka | 360.7 | 340.6 | 369.6 | 325.4 | 321.3 | 288.7 | 291.1 | 309.6 | 308.6 | 297.9 | 297.7 | 352.4 |
| Nagoya | 344.1 | 340.9 | 354.1 | 320.8 | 304.3 | 279.5 | 283.2 | 298.6 | 301.6 | 281.5 | 275.6 | 334.0 |
| Tsu | 379.0 | 397.9 | 375.7 | 335.6 | 315.4 | 279.7 | 288.1 | 324.0 | 304.8 | 297.8 | 301.9 | 361.4 |
| Otsu | 359.7 | 358.3 | 361.3 | 345.8 | 305.8 | 271.4 | 272.5 | 285.7 | 285.0 | 279.1 | 282.7 | 350.9 |
| Kyoto | 366.4 | 349.4 | 356.8 | 315.8 | 295.0 | 254.1 | 262.8 | 286.5 | 283.3 | 272.6 | 282.4 | 347.3 |
| Osaka | 321.1 | 316.8 | 329.1 | 294.0 | 278.7 | 252.3 | 264.8 | 297.3 | 284.1 | 269.5 | 259.6 | 307.4 |
| Kobe | 310.3 | 328.2 | 330.6 | 290.4 | 276.3 | 252.7 | 247.4 | 274.8 | 268.1 | 268.2 | 260.1 | 310.4 |
| Nara | 383.5 | 381.3 | 381.4 | 357.6 | 330.6 | 285.6 | 298.4 | 309.0 | 312.8 | 305.3 | 302.6 | 382.2 |
| Wakayama | 348.1 | 353.5 | 355.7 | 308.0 | 295.2 | 274.5 | 282.2 | 315.1 | 294.7 | 285.9 | 278.2 | 334.5 |
| Tottori | 350.9 | 362.6 | 358.1 | 308.1 | 277.7 | 251.8 | 245.3 | 265.6 | 256.9 | 252.9 | 261.2 | 330.2 |
| Matsue | 369.3 | 389.8 | 385.6 | 346.9 | 317.5 | 284.5 | 273.7 | 301.4 | 283.3 | 281.5 | 285.1 | 365.6 |
| Okayama | 340.2 | 339.9 | 357.8 | 319.3 | 285.9 | 258.9 | 265.4 | 288.2 | 285.2 | 269.6 | 268.4 | 318.2 |
| Hiroshima | 358.4 | 368.5 | 358.6 | 322.5 | 309.5 | 283.7 | 276.4 | 301.2 | 303.5 | 275.8 | 288.1 | 353.3 |
| Yamaguchi | 375.4 | 373.9 | 368.7 | 324.5 | 306.5 | 272.7 | 280.6 | 321.3 | 287.7 | 292.0 | 284.3 | 372.0 |
| Tokushima | 351.3 | 359.5 | 361.0 | 319.5 | 288.5 | 272.2 | 273.1 | 304.2 | 306.5 | 290.7 | 289.9 | 344.3 |
| Takamatsu | 372.2 | 364.1 | 377.1 | 345.3 | 305.3 | 268.1 | 295.9 | 331.2 | 333.5 | 310.7 | 301.8 | 381.0 |
| Matsuyama | 333.6 | 326.2 | 341.1 | 303.4 | 290.4 | 253.6 | 267.5 | 294.6 | 288.2 | 277.7 | 267.6 | 328.8 |
| Kochi | 358.5 | 361.4 | 362.9 | 326.1 | 305.5 | 285.9 | 285.2 | 307.9 | 300.5 | 295.1 | 289.8 | 345.0 |
| Fukuoka | 322.3 | 317.5 | 333.1 | 289.9 | 282.7 | 259.0 | 276.3 | 289.0 | 286.2 | 275.0 | 274.3 | 326.0 |
| Saga | 347.9 | 347.4 | 337.5 | 317.5 | 292.0 | 275.7 | 276.1 | 307.1 | 308.7 | 284.5 | 275.8 | 358.1 |
| Nagasaki | 332.4 | 336.2 | 333.6 | 302.9 | 272.6 | 245.1 | 269.5 | 290.1 | 284.9 | 275.8 | 270.3 | 327.0 |
| Kumamoto | 322.2 | 310.2 | 313.7 | 273.9 | 269.6 | 249.5 | 258.8 | 287.2 | 287.9 | 270.2 | 268.5 | 320.9 |
| Oita | 347.6 | 344.9 | 334.3 | 306.6 | 301.8 | 278.3 | 281.5 | 308.4 | 298.7 | 285.6 | 287.9 | 351.9 |
| Miyazaki | 306.5 | 290.9 | 296.0 | 270.8 | 255.5 | 256.6 | 250.6 | 272.7 | 284.2 | 265.7 | 266.4 | 312.6 |
| Kagoshima | 323.8 | 318.2 | 322.2 | 299.6 | 282.2 | 268.3 | 281.8 | 315.8 | 304.6 | 287.4 | 264.1 | 318.5 |
| Naha | 243.3 | 239.4 | 242.1 | 233.9 | 228.3 | 233.2 | 254.4 | 284.2 | 265.7 | 254.1 | 231.1 | 255.2 |

**Supplementary Table 2. Monthly GHG emissions of 47 prefectural-level cities during the pandemic (2020, kgCO_2_eq/cap/month)**

|  | January | February | March | April | May | June | July | August | September | October | November | December |
| --- | --- | --- | --- | --- | --- | --- | --- | --- | --- | --- | --- | --- |
| Sapporo | 451.0 | 471.6 | 464.4 | 462.0 | 396.3 | 358.0 | 298.1 | 318.9 | 328.9 | 324.4 | 366.3 | 440.3 |
| Aomori | 478.2 | 454.4 | 441.0 | 417.1 | 332.8 | 277.6 | 260.9 | 286.9 | 253.2 | 296.4 | 337.2 | 480.4 |
| Morioka | 411.8 | 421.6 | 400.2 | 401.9 | 328.5 | 273.0 | 268.3 | 276.1 | 273.4 | 264.2 | 298.9 | 393.2 |
| Sendai | 360.1 | 326.7 | 357.6 | 289.4 | 288.8 | 263.2 | 286.5 | 270.0 | 238.2 | 274.4 | 281.0 | 331.5 |
| Akita | 415.8 | 439.7 | 423.0 | 411.1 | 341.8 | 309.6 | 315.5 | 298.2 | 315.8 | 364.6 | 383.1 | 484.0 |
| Yamagata | 449.7 | 439.4 | 439.3 | 378.5 | 318.4 | 301.7 | 265.4 | 266.0 | 265.9 | 316.0 | 318.7 | 380.3 |
| Fukushima | 394.9 | 407.5 | 395.6 | 414.7 | 327.1 | 334.3 | 276.2 | 268.2 | 258.2 | 329.6 | 298.8 | 365.3 |
| Mito | 364.4 | 364.5 | 350.8 | 335.6 | 337.4 | 298.2 | 279.2 | 285.9 | 309.6 | 273.8 | 311.5 | 337.0 |
| Utsunomiya | 343.3 | 337.5 | 335.7 | 296.4 | 291.9 | 283.1 | 272.9 | 288.6 | 273.5 | 314.5 | 303.7 | 330.0 |
| Maebashi | 358.3 | 364.6 | 360.5 | 340.8 | 294.8 | 320.6 | 300.8 | 346.5 | 316.4 | 290.3 | 271.0 | 310.1 |
| Saitama | 328.0 | 327.6 | 365.0 | 343.6 | 329.4 | 358.6 | 292.0 | 265.6 | 293.9 | 277.5 | 311.6 | 319.1 |
| Chiba | 319.5 | 304.1 | 316.8 | 279.5 | 306.6 | 274.8 | 262.8 | 280.8 | 303.2 | 287.5 | 293.3 | 309.5 |
| Tokyo | 314.3 | 340.4 | 351.3 | 314.9 | 293.0 | 283.1 | 282.6 | 288.1 | 286.3 | 307.5 | 311.8 | 335.0 |
| Yokohama | 344.5 | 337.8 | 361.6 | 330.3 | 285.0 | 286.8 | 285.3 | 274.0 | 283.4 | 275.1 | 272.8 | 306.8 |
| Niigata | 332.4 | 348.9 | 352.0 | 293.9 | 278.4 | 319.0 | 269.3 | 293.3 | 292.1 | 292.4 | 282.2 | 345.5 |
| Toyama | 356.5 | 371.4 | 392.8 | 367.7 | 325.3 | 308.2 | 294.5 | 310.2 | 279.7 | 308.2 | 300.8 | 375.0 |
| Kanazawa | 354.7 | 372.4 | 405.2 | 335.4 | 286.5 | 287.9 | 281.4 | 289.6 | 297.0 | 267.6 | 268.5 | 316.8 |
| Fukui | 361.1 | 369.9 | 364.9 | 341.0 | 342.4 | 329.8 | 314.7 | 315.1 | 303.9 | 284.6 | 293.7 | 323.0 |
| Kofu | 339.7 | 338.9 | 374.6 | 309.0 | 271.8 | 316.1 | 260.5 | 279.2 | 295.4 | 312.6 | 305.0 | 326.2 |
| Nagano | 388.0 | 393.6 | 387.9 | 312.2 | 301.6 | 288.6 | 266.9 | 285.0 | 297.2 | 280.4 | 305.9 | 388.1 |
| Gifu | 372.0 | 396.3 | 428.0 | 335.0 | 359.6 | 308.8 | 272.2 | 284.0 | 300.6 | 273.6 | 300.9 | 316.6 |
| Shizuoka | 298.1 | 319.4 | 331.0 | 317.0 | 283.6 | 254.1 | 312.0 | 306.2 | 327.3 | 292.6 | 288.1 | 308.8 |
| Nagoya | 318.4 | 325.7 | 320.1 | 330.0 | 281.2 | 258.9 | 254.8 | 261.9 | 284.5 | 258.4 | 265.1 | 328.9 |
| Tsu | 333.6 | 373.0 | 356.4 | 309.0 | 268.4 | 255.4 | 251.0 | 275.0 | 287.5 | 300.9 | 307.5 | 302.5 |
| Otsu | 337.8 | 303.9 | 298.2 | 277.3 | 309.1 | 268.6 | 242.2 | 282.9 | 366.7 | 300.8 | 265.6 | 329.8 |
| Kyoto | 325.1 | 308.9 | 301.3 | 280.2 | 262.7 | 247.5 | 243.5 | 289.7 | 291.4 | 262.3 | 264.6 | 294.0 |
| Osaka | 270.7 | 300.9 | 315.4 | 288.9 | 262.8 | 257.8 | 258.2 | 265.1 | 265.6 | 282.6 | 239.9 | 279.1 |
| Kobe | 257.2 | 292.6 | 332.7 | 277.5 | 247.4 | 256.6 | 278.9 | 274.2 | 268.0 | 271.2 | 266.0 | 301.8 |
| Nara | 334.6 | 358.8 | 379.0 | 330.3 | 341.5 | 286.0 | 295.8 | 331.7 | 326.5 | 349.5 | 314.3 | 368.3 |
| Wakayama | 307.5 | 318.5 | 335.4 | 286.5 | 257.7 | 238.1 | 249.9 | 252.2 | 287.9 | 287.4 | 262.5 | 295.9 |
| Tottori | 338.8 | 364.9 | 383.5 | 327.7 | 254.2 | 258.6 | 239.1 | 265.0 | 312.0 | 263.9 | 260.4 | 291.8 |
| Matsue | 347.9 | 384.1 | 351.6 | 311.9 | 317.8 | 313.7 | 272.3 | 315.6 | 272.9 | 272.0 | 274.5 | 339.4 |
| Okayama | 283.9 | 300.5 | 313.6 | 267.1 | 249.8 | 239.6 | 230.3 | 239.6 | 276.4 | 267.6 | 253.7 | 279.7 |
| Hiroshima | 328.6 | 342.3 | 332.4 | 309.8 | 298.3 | 295.8 | 274.5 | 303.2 | 302.9 | 293.5 | 256.8 | 348.1 |
| Yamaguchi | 335.1 | 329.7 | 349.3 | 317.6 | 292.4 | 292.0 | 274.2 | 289.5 | 282.5 | 258.8 | 269.0 | 307.6 |
| Tokushima | 333.9 | 332.1 | 363.4 | 315.4 | 293.2 | 286.7 | 273.7 | 303.5 | 327.3 | 299.7 | 304.8 | 357.3 |
| Takamatsu | 332.8 | 333.9 | 339.2 | 287.6 | 258.5 | 256.1 | 266.5 | 260.1 | 278.4 | 262.5 | 259.0 | 324.2 |
| Matsuyama | 331.9 | 299.2 | 334.2 | 256.2 | 263.0 | 240.8 | 220.3 | 240.6 | 257.3 | 256.0 | 249.9 | 280.6 |
| Kochi | 309.1 | 313.3 | 311.5 | 283.3 | 284.8 | 279.8 | 266.2 | 304.1 | 284.0 | 269.9 | 270.6 | 295.8 |
| Fukuoka | 295.8 | 296.8 | 301.4 | 289.2 | 261.9 | 304.6 | 271.1 | 322.5 | 294.3 | 297.1 | 310.5 | 292.4 |
| Saga | 334.1 | 369.6 | 341.9 | 322.7 | 286.9 | 304.7 | 267.8 | 318.2 | 307.5 | 280.6 | 273.0 | 330.5 |
| Nagasaki | 305.3 | 327.5 | 295.7 | 281.8 | 269.1 | 275.1 | 241.9 | 276.8 | 306.2 | 269.5 | 252.3 | 308.3 |
| Kumamoto | 258.9 | 301.1 | 292.3 | 250.0 | 262.9 | 249.9 | 280.2 | 261.2 | 270.0 | 263.4 | 258.6 | 294.6 |
| Oita | 288.7 | 291.0 | 329.0 | 276.2 | 264.0 | 288.2 | 281.0 | 317.1 | 286.2 | 265.1 | 271.5 | 285.3 |
| Miyazaki | 279.9 | 313.6 | 301.2 | 269.7 | 272.0 | 236.4 | 243.3 | 273.9 | 253.2 | 264.0 | 243.5 | 303.6 |
| Kagoshima | 302.8 | 304.7 | 333.4 | 283.4 | 256.3 | 250.6 | 242.1 | 282.7 | 276.2 | 298.3 | 280.0 | 309.1 |
| Naha | 225.2 | 234.6 | 222.3 | 216.1 | 208.7 | 223.6 | 253.3 | 269.5 | 262.3 | 267.0 | 237.6 | 245.8 |

**Supplementary References**

1 Natural Resources Canada. *Learn the facts: Cold weather effects on fuel efficiency*, <https://www.nrcan.gc.ca/energy-efficiency/transportation-alternative-fuels/personal-vehicles/choosing-right-vehicle/tips-buying-fuel-efficient-vehicle/factors-affect-fuel-efficiency/cold-weather/21032> (2018).

2 Alvarez, R. & Weilenmann, M. Effect of low ambient temperature on fuel consumption and pollutant and CO2 emissions of hybrid electric vehicles in real-world conditions. *Fuel* **97**, 119-124 (2012).

3 Fujiwara, Y., Enai, M., Suzuki, K. & Hayama, H. Survey on the actual operation and the performance of central heating system for highly insulated and airtight houses in hokkaido. *Journal of Environmental Engineering (Transactions of AIJ)* **73**, 767-774 (2008).

4 Office for Covid and Other Emerging Infectious Disease Control. Basic Policies for Novel Coronavirus Disease Control by the Government of Japan (ed Government of Japan Cabinet Secretariat) <<https://corona.go.jp/en/news/news_20200510_77.html>> (2020).

5 Kurita, J., Sugawara, T. & Ohkusa, Y. The second COVID-19 emergency status declaration for Japan: effects as of February 7, 2021. *medRxiv*, 2020.2012. 2029.20248977 (2021).

6 Cabinet Public Affairs Office. Declaration of a State of Emergency in response to the Novel Coronavirus Disease (February 26) (ed Cabinet Secretariat) <<https://japan.kantei.go.jp/ongoingtopics/_00043.html>> (2021).

7 Cabinet Public Affairs Office. Declaration of a State of Emergency in response to the Novel Coronavirus Disease (January 7) (ed Cabinet Secretariat) <<https://japan.kantei.go.jp/ongoingtopics/_00038.html>> (2021).

8 Office for COVID-19 and Other Emerging Infectious Disease Control. *Change in restrictions with the lift of the State of Emergency*, <https://corona.go.jp/en/emergency/> (2021).

9 Chappell, B. *Japan Declares 3rd State Of Emergency, 3 Months Ahead Of Olympics*, <https://www.npr.org/sections/coronavirus-live-updates/2021/04/23/990133421/japan-declares-3rd-state-of-emergency-3-months-ahead-of-olympics> (2021).
