# Supplementary material for: Japanese urban household carbon footprints during early-stage COVID-19 pandemic were consistent with those over the past decade
Source: NPJ Urban Sustain. 2023 Mar 29;3(1):19. doi: 10.1038/s42949-023-00095-z (PMC10052282; doi:10.1038/s42949-023-00095-z)
Supplement: Supplementary file 2 — Reporting Summary [file 42949_2023_95_MOESM2_ESM.pdf]

nature portfolio

Corresponding author(s): Yin Long, Liqiao Huang, Zhifu Mi

Last updated by author(s): Feb 17, 2023

Reporting Summary

Nature Portfolio wishes to improve the reproducibility of the work that we publish. This form provides structure for consistency and transparency in reporting. For further information on Nature Portfolio policies, see our Editorial Policies and the Editorial Policy Checklist.

Statistics

For all statistical analyses, confirm that the following items are present in the figure legend, table legend, main text, or Methods section.

n/a	Confirmed	
☐	☒	The exact sample size (*n*) for each experimental group/condition, given as a discrete number and unit of measurement
☐	☒	A statement on whether measurements were taken from distinct samples or whether the same sample was measured repeatedly
☐	☒	The statistical test(s) used AND whether they are one- or two-sided<br>*Only common tests should be described solely by name; describe more complex techniques in the Methods section.*
☐	☒	A description of all covariates tested
☐	☒	A description of any assumptions or corrections, such as tests of normality and adjustment for multiple comparisons
☐	☒	A full description of the statistical parameters including central tendency (e.g. means) or other basic estimates (e.g. regression coefficient) AND variation (e.g. standard deviation) or associated estimates of uncertainty (e.g. confidence intervals)
☒	☐	For null hypothesis testing, the test statistic (e.g. *F*, *t*, *r*) with confidence intervals, effect sizes, degrees of freedom and *P* value noted<br>*Give P values as exact values whenever suitable.*
☒	☐	For Bayesian analysis, information on the choice of priors and Markov chain Monte Carlo settings
☒	☐	For hierarchical and complex designs, identification of the appropriate level for tests and full reporting of outcomes
☒	☐	Estimates of effect sizes (e.g. Cohen's *d*, Pearson's *r*), indicating how they were calculated

Our web collection on statistics for biologists contains articles on many of the points above.

Software and code

Policy information about availability of computer code

Data collection	The input–output lifecycle inventory dataset (3EID) and the Family Income and Expenditure Survey (FIES) dataset provide information on household consumption and the Greenhouse gas emission. The FIES survey selected approximately 9,000 households from appropriate households (excluding households such as single student occupants, hospital inpatients, foreign households, etc.). In order to avoid bias in the obtained numbers and to free the sampled households from the burden of long-term bookkeeping, the sample was updated periodically. A weekly survey on retail prices at filling stations conducted by the Ministry of Economy, Trade, and Industry of Japan provides retail prices for gasoline and kerosene each week.
Data analysis	We analyzed data obtained from the aforementioned approaches with all sources attached in the manuscript.

For manuscripts utilizing custom algorithms or software that are central to the research but not yet described in published literature, software must be made available to editors and reviewers. We strongly encourage code deposition in a community repository (e.g. GitHub). See the Nature Portfolio guidelines for submitting code & software for further information.

Data

Policy information about availability of data

All manuscripts must include a data availability statement. This statement should provide the following information, where applicable:

- Accession codes, unique identifiers, or web links for publicly available datasets
- A description of any restrictions on data availability
- For clinical datasets or third party data, please ensure that the statement adheres to our policy

All data aggregated or analyzed in the current study are available from the corresponding author on reasonable request. In this research, weekly retail prices for fuels are from the Ministry of Economy, Trade, and Industry of Japan (https://www.enecho.meti.go.jp/statistics/petroleum_and_lpgas/pl007/results.html), the Agency of Natural Resource and Energy (https://www.enecho.meti.go.jp/en/category/whitepaper/), and Oil Information Center at the Institute of Energy Economics (https://oil-info.ieej.or.jp/price/price.html.). Detailed information on household expenditures of Japan is from FIES (https://www.stat.go.jp/english/data/kakei/index.html). Emission intensity data are from Japan's Ministry of the Environment (https://ghg-santeikohyo.env.go.jp/calc) and Economic and Social Research Institute, Cabinet Office of Japan (https://www.esri.cao.go.jp/en/sna/menu.html).

Human research participants

Policy information about studies involving human research participants and Sex and Gender in Research.

Reporting on sex and gender	Sex and gender are not discussed in this study.
Population characteristics	Cover the population in 47 prefectural-level cities in japan.
Recruitment	No recruitment involved in this study.
Ethics oversight	No ethics oversight included in this study.

Note that full information on the approval of the study protocol must also be provided in the manuscript.

Field-specific reporting

Please select the one below that is the best fit for your research. If you are not sure, read the appropriate sections before making your selection.

☐ Life sciences ☐ Behavioural & social sciences ☒ Ecological, evolutionary & environmental sciences

For a reference copy of the document with all sections, see nature.com/documents/nr-reporting-summary-flat.pdf

Ecological, evolutionary & environmental sciences study design

All studies must disclose on these points even when the disclosure is negative.

Study description	The present study first generates city-level carbon footprint data from 2011 to 2021 to quantify both the direct and indirect city-level carbon footprints and takes 47 Japanese cities as an example. The cities selected in this study are the capital cities of 43 prefectures, two urban prefectures (Osaka and Kyoto), one territory (Hokkaidō), and the metropolis Tokyo, the population of which accounts for more than 50% of Japan's population. Approximately 500 household consumption items (by month/city) were captured with their embodied carbon footprint, covering citizens' major living demands (e.g., food, home energy, accommodation, and transportation). In addition, the emission variations from January 2020 were extracted, with a special focus on revealing how the consumption behaviors of city residents were impacted in response to COVID-19. The findings provide important insights for evaluating the environmental consequences of citizen behaviors under the abrupt impact of unexpected social events.
Research sample	A total of 395 items were included in the emission intensity dataset 3EID in 2011 and 390 items in 2015. Based on the results obtained through cross-mapping the 3EID datasets with the corresponding FIES datasets, we obtained an emission inventory with 495 items between 2011 and 2014, 512 items between 2015 and 2019, and 504 items between 2020 and 2021.
Sampling strategy	Due to the differences between the 3EID database's industry classifications and consumption elements in the FIES expenditure data, this study rematched the data according to a method described elsewhere and sketched out a feasible approach for acquiring data for the year 2015 in one of the supporting documents. Thus, linear interpolation is applied to assess the indirect emission intensities for all study items for the relevant years, thereby obtaining the values.
Data collection	In this research, weekly retail prices for fuels are from the Ministry of Economy, Trade, and Industry of Japan (https://www.enecho.meti.go.jp/statistics/petroleum_and_lpgas/pl007/results.html), the Agency of Natural Resource and Energy (https://www.enecho.meti.go.jp/en/category/whitepaper/), and Oil Information Center at the Institute of Energy Economics (https://oil-info.ieej.or.jp/price/price.html.). Detailed information on household expenditures of Japan is from FIES (https://www.stat.go.jp/english/data/kakei/index.html). Emission intensity data are from Japan's Ministry of the Environment (https://ghg-

	santeikohyo.env.go.jp/calc) and Economic and Social Research Institute, Cabinet Office of Japan (https://www.esri.cao.go.jp/en/sna/menu.html).
Timing and spatial scale	Monthly average carbon emissions in 47 Japanese cities from January 2011 to June 2021
Data exclusions	The FIES survey selected approximately 9,000 households from appropriate households (excluding households such as single student occupants, hospital inpatients, foreign households, etc.). In order to avoid bias in the obtained numbers and to free the sampled households from the burden of long-term bookkeeping, the sample was updated periodically.
Reproducibility	All attempts to repeat the experiment were successful.
Randomization	Not relevant to this study. This study is a qualitative analysis of household carbon footprint, which is based on existing household expenditure data rather than randomization.
Blinding	Not relevant to this study. This study is a qualitative analysis of household carbon footprint, which is based on existing household expenditure data rather than Blinding.

Did the study involve field work? ☐ Yes ☒ No

Reporting for specific materials, systems and methods

We require information from authors about some types of materials, experimental systems and methods used in many studies. Here, indicate whether each material, system or method listed is relevant to your study. If you are not sure if a list item applies to your research, read the appropriate section before selecting a response.

Materials & experimental systems

n/a	Involved in the study
☒	☐ Antibodies
☒	☐ Eukaryotic cell lines
☒	☐ Palaeontology and archaeology
☒	☐ Animals and other organisms
☒	☐ Clinical data
☒	☐ Dual use research of concern

Methods

n/a	Involved in the study
☒	☐ ChIP-seq
☒	☐ Flow cytometry
☒	☐ MRI-based neuroimaging
